# Microbial Communities of the Shallow-Water Hydrothermal Vent Near Naples, Italy, and Chemosynthetic Symbionts Associated With a Free-Living Marine Nematode

**DOI:** 10.3389/fmicb.2020.02023

**Published:** 2020-08-20

**Authors:** Laure Bellec, Marie-Anne Cambon-Bonavita, Lucile Durand, Johanne Aube, Nicolas Gayet, Roberto Sandulli, Christophe Brandily, Daniela Zeppilli

**Affiliations:** ^1^Ifremer, Centre Brest, REM/EEP/LEP, ZI de la Pointe du Diable, CS10070, Plouzané, France; ^2^Laboratoire de Microbiologie des Environnements Extrêmes, Ifremer, CNRS, Univ Brest, Plouzané, France; ^3^EPOC, UMR 5805, University of Bordeaux, Arcachon, France; ^4^Laboratory of Marine Ecology, Department of Science and Technology, University of Naples “Parthenope,” Naples, Italy

**Keywords:** nematode, shallow-water hydrothermal vent, sulfur-oxidizing bacteria, iron cycle, *Zetaproteobacteria*

## Abstract

Shallow-water hydrothermal vents are widespread, especially in the Mediterranean Sea, owing to the active volcanism of the area. Apart free microbial communities’ investigations, few biological studies have been leaded yet. Investigations of microbial communities associated with Nematoda, an ecologically important group in sediments, can help to improve our overall understanding of these ecosystems. We used a multidisciplinary-approach, based on microscopic observations (scanning electron microscopy: SEM and Fluorescence *In Situ* Hybridization: FISH) coupled with a molecular diversity analysis using metabarcoding, based on the 16S rRNA gene (V3-V4 region), to characterize the bacterial community of a free-living marine nematode and its environment, the shallow hydrothermal vent near Naples (Italy). Observations of living bacteria in the intestine (FISH), molecular and phylogenetic analyses showed that this species of nematode harbors its own bacterial community, distinct from the surrounding sediment and water. Metabarcoding results revealed the specific microbiomes of the sediment from three sites of this hydrothermal area to be composed mainly of sulfur oxidizing and reducing related bacteria.

## Introduction

Shallow-water hydrothermal vents (<200 m in depth) have a widespread biogeographical distribution, generally associated with active submarine volcanism, and can be considered as intermediate environments between deep-sea and terrestrial hydrothermal systems (Tarasov et al., 2005). They are characterized by a fluid temperature range of 10–119°C (Tarasov et al., 2005). Many shallow-water hydrothermal vents show high concentrations of compounds and metal elements involved in geochemical cycles, including carbon, sulfur, methane, and iron. Due to these extreme conditions, shallow-water vents are outstanding environments to investigate the diversity of microorganisms involved in organic matter synthesis, breakdown or mineralization, metal cycles and interactions with fauna. Despite relatively easy access to these sites, microbiology of shallow-water hydrothermal vents is still in its infancy compared with less accessible deep-sea ones. Furthermore, primary production at shallow-water hydrothermal vents is based on two parallel systems: photosynthesis and chemosynthesis. The contribution of chemosynthesis to primary production is highly variable depending of the kind of vent emission, with for instance 1% at Kraternaya Bight to 50% at Matupi Harbor (Tarasov et al., 1999). At the shallow hydrothermal vents of Panarea Island (Italy), the primary production is supported by a complex of phototroph and chemolithotroph microbial communities (Maugeri et al., 2009, 2010b). Maugeri et al. (2009) characterized the microbial communities at shallow hydrothermal vents differing in depth and temperature using an approach mixing culture and molecular methods (DGGE). Sulfur-oxidizing bacteria were detected and bacterial abundance was highest in the warmest site (Maugeri et al., 2009). Bacteria affiliated to *Gammaproteobacteria* and members of the *Chromatiaceae* were observed at the highest temperature and the lowest pH (Maugeri et al., 2010b). Studies at shallow hydrothermal vents suggest parallel presence of photosynthetic and chemolithotrophic primary production, embodying the main difference between shallow and deep-sea hydrothermal vents. At shallow depth, diverse types of biological mats can be observed (diatom, algal-bacterial and microbial). At west Pacific and Mediterranean shallow-water vents, many chemosynthetic bacteria involved in the sulfur cycle were reported in the algae-bacterial or microbial mats, as were thermophilic bacteria and iron-reducing bacteria (Tarasov et al., 2005). Chemosynthetic symbioses at shallow vents have, so far, generally been reported with bivalve and polychaete hosts (Dubilier et al., 2008). Meiofaunal organisms, which are small benthic invertebrates, are well adapted to extreme conditions and could represent a significant part of the total abundance and diversity of hydrothermal vent fauna (Zeppilli et al., 2018). Vent meiofauna is mainly composed of copepods and nematodes, especially the family Onchalaimidae, which has been reported multiple times close to emission points on diverse shallow hydrothermal vents (Dando et al., 1995; Thiermann et al., 1995; Zeppilli and Danovaro, 2009). They were also found to be present in sedimentary environments such as harbors (Bellec et al., 2019) and are associated with bivalve byssuses in the deep sea (Bellec et al., 2018). These recent studies revealed potential symbiotic associations of nematodes with sulfur-oxidizing bacteria affiliated to *Campylobacterota* (previously known as *Epsilonproteobacteria*) (Waite et al., 2017, 2018) and *Gammaproteobacteria*, especially with a high degree of ectosymbiosis in the harbor. Additionally, detection of the *AprA* gene, involved in sulfur metabolism in the nematode microbial communities, suggests that chemosynthesis could be a mechanism closely associated with nematodes in shallow water (Bellec et al., 2019).

In the Mediterranean Sea, several shallow-water hydrothermal vents have been identified resulting from the collision of the African and European plates. These vent environments are characterized by major compounds such as carbon dioxide, sulfur dioxide, hydrogen sulfide, methane and hydrogen (Dando et al., 1999). The Gulf of Naples (Italy) provides access to a shallow vent, thus enabling microbiological investigations. A gas-rich hydrothermal fluid with acidification of pH was reported at a few kilometers away from Ischia island and named “Secca delle Fumose” (Di Napoli et al., 2016). This shallow submarine relief was investigated for extremophile microorganisms potentially valuable in biotechnology, with culture trials but no study of overall microbial diversity (Maugeri et al., 2010a), and very recently for its macrofaunal community (Donnarumma et al., 2019). To our knowledge, this is the first complete study dealing with sediment and seawater microbial diversity together with nematode-associated microbial communities.

In this study, we investigated both the environmental microbial community and the microbiome of the dominant species of nematode sampled in the “Secca delle Fumose” active hydrothermal vent field, Gulf of Naples (Italy). Using molecular together with microscopy approaches, we pursued several objectives. Firstly, we studied closely-spaced locations around a small geyser to see whether they had different microbial communities. Then we investigated if nematodes had their own bacterial communities that would differ from their surrounding habitats, which may suggest a possible symbiosis. Finally, we examined whether these associated bacterial communities could play a role in the sulfur or other cycles.

## Materials and Methods

### The Study Area and Sample Collection

**FIGURE 1:**
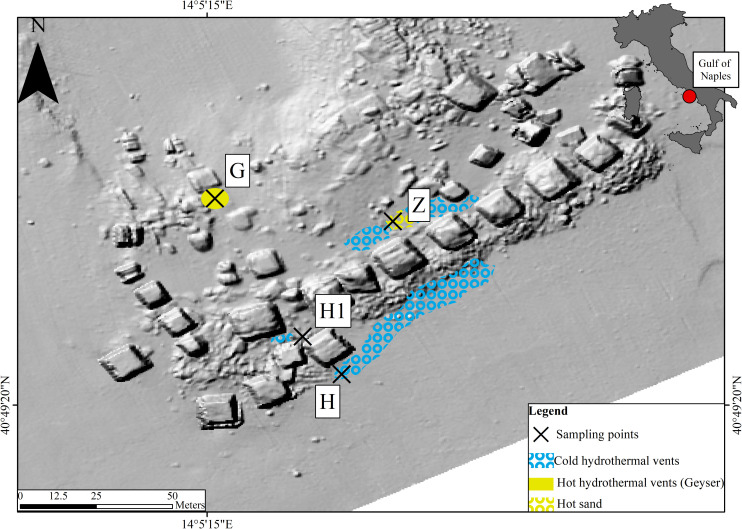
Map of the Gulf of Naples showing locations of the sampling stations.

The “Secca delle Fumose” study area is located within the largest degassing structure in the Campi Flegrei caldera offshore sector, at about 800 m off the coastline of the north-western part of Pozzuoli Bay (Gulf of Naples, Italy) (40°49′23″ N; 14°05′15″ E) (Tedesco et al., 1990; Passaro et al., 2013; Di Napoli et al., 2016). Morpho-bathymetric data and archeological surveys show that this shoal is largely of anthropogenic origin, consisting of an aggregation of 26 pillars from the Roman age (first century BC), with a height of 7 m, mostly standing on a seafloor at about 12 m depth where hydrothermal vents occur, and covering area of about 36 m (9 m × 4 sides). Within this area, four sampling sites were selected (Figure 1): two hydrothermal vents (H and H1) with associated sediment temperatures around 25°C, characterized by the occurrence of white bacterial mats; a third site with a water geyser (emitting water up to 72°C), characterized by yellow depositions (G); and a fourth site characterized by hot sediment (around 60°C) and yellowish sand (Z). Environmental parameters (i.e., water temperature, sediment temperature, depth, and interstitial water chemistry), water (three replicates), the top layer of sediment (2–3 cm) (three replicates), and sediment with inhabiting meiofauna were sampled during a single dive at site G (October 12 2016) and a unique dive at the four sites (November 1 2017) (Supplementary Table S1). With scuba-diving manipulations, it was not practically possible to sample the fluid directly or take sediment samples from inside the geyser. Therefore, site G samples were collected as close as possible (< 30 cm) to the geyser opening. Each sediment sample (top layer) was collected using sterile Falcon tubes (50 ml), frozen, and kept at -20°C until DNA extraction. Seawater samples were collected just above each sediment site using 1-L sterile jars. Each seawater sample was filtered by gravity (filters 0.22μm) in the laboratory on the same day and filters were frozen at -20°C until DNA extraction. Sediment and seawater samples were kept at 4°C during transport from the study area to the laboratory (less than 2 h). For the nematode community, samples were collected by scuba-diving operators using an air-lift suction sampler equipped with a 1-mm nylon mesh bag within a 50 × 50 cm frame, reaching a depth of 10 cm into the sediment. Sediment and water temperatures were measured *in situ* using an underwater thermometer.

### Chemical Analyses

Push cores with a diameter of 5 cm were retrieved manually by divers on precisely selected habitats. Pore waters were then collected from cores using Rhizon samplers (Rhizosphere Research Products R.V., Wageningen), which are thin rods covered by a hydrophilic porous polymer designed to extract water from sediment with a vacuum (Seeberg-Elverfeldt et al., 2005). Rhizon samplers, 5 cm in length, were horizontally inserted into predrilled holes in the core liner, which were sealed during core retrieval. To minimize the risk of interference between adjacent samplers, 6-mL water samples were collected every 2 cm along the cores. One 4-mL aliquot was preserved using 20 μL of a saturated HgCl_2_ solution and transferred into a N_2_-purged glass vial closed with a septum for CH_4_ analyses, another 1 mL aliquot was preserved using ZnCl_2_ (saturated) for H_2_S analysis, and a last 1-mL aliquot was acidified with 2 μL of 69% nitric acid for SO_4_^2–^ analysis. All these chemical aliquots were stored at 4°C prior to analysis.

Methane concentrations were measured by headspace gas chromatography using a PR2100 gas chromatograph equipped with a flame ionization detector (GC/FID Perichrom, France) connected to a headspace autosampler (dani HSS 86.50) (Sarradin and Caprais, 1996). The quantification limit of this method is 0.1 μmol.L^–1^ and the relative standard deviation of the method is 3%. Sulfates were measured with an ion-exchange chromatograph DionexICS-5000 (Thermo Scientific^®^), equipped with an Ionpac AS22-SC column, an Ionpac AG 22-SC precolumn and an electrical conductivity detector. The eluent was composed of 5 mM Na_2_CO_3_ and 1.75 mM NaHCO_3_. IAPSO standard seawater was used as a certified reference material for calibration. Sulfides were determined in the laboratory by colorimetry using the methylene blue method (Cline, 1969).

### Nematode Sorting and Fixation

To verify that all nematodes belonged to the same species, specimens were sorted under a stereomicroscope (M125; Leica, Wetzlar, Germany) and we made a morphological examination. A set of specimens was immediately frozen at -80°C for later molecular analyses (on both nematodes and microbial diversity). Other specimens were stored for Scanning Electron Microscopy (SEM) studies: these nematodes were fixed in glutaraldehyde 2.5% for 16 h at 4°C then transferred to a sodium azide solution (0.065 g in 150 ml filtered sea-water) and stored at 4°C until use. Another set of nematodes was stored for FISH analyses: samples were fixed for 2 h in 3% formaldehyde-sterile seawater solution and rinsed with 1X phosphate-buffered saline (PBS)–sterile seawater solution (1:1). These samples were stored in absolute ethanol-2X PBS solution (1:1) at -20°C until use (Durand et al., 2010).

### Nematode DNA Extraction, PCR, and Sequencing

Species assignment of the nematodes directly frozen at -80°C was verified with a molecular approach (Table 1). Total DNA was extracted individually from each whole nematode, using the Qiagen^®^ DNeasy Blood & Tissue kit following manufacturer’s instructions. Partial fragments of 18S rRNA (597 bp), and the 28S rRNA gene (654 bp) were amplified with different primer pairs (Supplementary Table S2). Ribosomal (18S and 28S) amplifications were performed on a Gene-Amp^™^ PCR system 9700 thermocycler (Applied Biosystems, Forster City, CA, United States) in a final volume of 25 μL, using the following mix: 2 μL extracted DNA were added to 5 μL 5X PCR buffer, 5 mM of each dNTP, 50 mM MgCl_2_, 20 μM of each of the two primers and 0.1 μL Taq polymerase (5 U/μL - Promega). The PCR cycles were 2 min at 94°C followed by 30 cycles of 1 min denaturation at 94°C, 1 min annealing at 55°C and 2 min extension at 72°C, with a final 10 min extension at 72°C. All amplification products were run on a 0.8% agarose-TAE gel to verify the size of the amplicons. Purification and Sanger sequencing of PCR products were performed by Macrogen^1^. Chromatograms were assembled and edited using Geneious 8.1.9 (Kearse et al., 2012)^2^ and all nucleotide differences were checked visually.

**TABLE 1:** Summary of experiments used to characterize and explore the bacterial diversity.

		G-2016 (*n*)	G (*n*)	H (*n*)	Z (*n*)	H1 (*n*)	Total
*Oncholaimus* sp.							
	18S	4	1	1	1	1	8
	28S	4	8	15	6	11	44
Bacterial diversity (16S rRNA)							
	*O* sp.	4	8	15	6	11	44
	Sediment	3	2	3	3	0	11
	Water	0	3	3	3	0	9
FISH		13					13
SEM		4					4

**n = number of samples.**

### 16S rRNA Bacterial Diversity Analyses by Illumina MiSeq

DNA from 44 specimens (based on morphological and genetic identification) from the four stations (G, H, H1, and Z) were sent to MR DNA (Shallowater, TX, United States^3^) for amplification of prokaryotic diversity based on 16S rRNA sequences (Table 1). DNA from the sediment and water were also used as environmental references. Total DNA was extracted from sediment (first layer) using the Qiagen^®^ DNeasy PowerMax Soil kit and from water using the Qiagen^®^ DNeasy PowerWater kit following the manufacturer’s instructions. Three negative controls (one from each extraction: nematode, sediment, water) were also used for amplification.

Sequencing was performed on a 450-bp fragment of the 16S rRNA gene using Illumina MiSeq technology. Briefly, the 16S V3-V4 variable region (primers 341/785, with barcode on the forward primer) (Klindworth et al., 2013) was put in a 28-cycle PCR using the HotStarTaq Plus Master Mix Kit (Qiagen, United States) under the following conditions: 94°C for 3 min, followed by 28 cycles of 94°C for 30 s, 53°C for 40 s and 72°C for 1 min, after which a final elongation step at 72°C for 5 min was performed. Multiple individual nematodes were pooled together in equal proportions based on their molecular weight and DNA concentrations. Pooled samples were purified using calibrated Ampure XP beads. The pooled and purified PCR product was then used to prepare a DNA library following the Illumina TruSeq DNA library preparation protocol. Sequencing was performed at MR DNA on a MiSeq sequencer following using 2 × 300 bp chemistry.

### Bioinformatics Data Processing

Prokaryotic 16S rRNA paired-end reads were merged using USEARCH (Edgar and Flyvbjerg, 2015) after q25 trimming of the ends. The resulting 16S reads were processed using the Find Rapidly OTU with the Galaxy Solution (FROGS) v2 pipeline (Escudié et al., 2018). In short, sequences were depleted of barcode, then sequences < 380 bp and those containing an ambiguous base were removed. Next, reads were clustered into *de novo* operational taxonomic units (OTUs) using Swarm (Mahé et al., 2014), with an aggregation distance equal to 3. Chimera were then removed with VSEARCH (Rognes et al., 2016). Additionally, a filter (for abundance) was applied to the OTUs, with an optimal threshold of 0.005% (Bokulich et al., 2013). The OTUs finally selected were taxonomically assigned by BLASTn + (Camacho et al., 2009) using the Silva release 132 reference database (Quast et al., 2013). Finally, filtrations were performed on BLAST taxonomic affiliation, with a minimum coverage of 60% and a minimum identity of 60%.

### Statistical Analysis

All statistical analyses and data visualization were carried out in R v3.6.2. Alpha diversity was computed using the Phyloseq (McMurdie and Holmes, 2013) and Vegan package (Oksanen et al., 2008) with Shannon and Pielou’s evenness metrics. We also calculated the observed richness on rarefied data (subsampled to 11697 sequences, the size of the smallest library). Difference in the alpha diversity indexes among conditions were tested using Kruskal-Wallis test followed by pairwise Wilcoxon tests; *p* < 0.05 was considered the threshold significance for a difference between conditions. Beta diversity analyses were performed on Bray-Curtis distances on rarefied dataset and were then visualized using PCoA. An analysis of betadispersion was used to quantify community variations for each Envtype. Difference in the distance to centroid among conditions was statistically tested by Kruskal-Wallis test followed by pairwise Wilcoxon tests. Sample groups were compared by a permutational multivariate analysis of variance (9999 permutations) with adonis function of the Vegan package. Multilevel comparisons for the conditions were also performed with the pairwise adonis function (Martinez Arbizu, 2017). Differences of taxa abundances associated to envtype and station were studied using a model based on negative binomial distribution as implemented by the DESeq function in the DESeq2 package. An adjusted *p* < 0.01 was considered significant. Only taxa accounting for more 1% of the overall relative abundance and overabundant were reported (log2FoldChange > 0). Boxplots, bubbleplots and scatterplot were produced with ggplot2. Venn diagrams were produced using Venny v2.1 software^4^.

### Scanning Electron Microscopy Observations

Four specimens from station G (2016) were chosen for SEM observations to analyze the presence of prokaryotes on the cuticle of the animals. After fixation, nematodes were postfixed in 0.8% osmium tetroxide for 20 h at 4°C and then dehydrated through an ethanol series. The nematodes were desiccated with a critical-point dryer (CPD 300; Leica, Wetzlar, Germany) and then mounted on a specimen stub. They were gold-coated using an SCD 040 (Blazers Union, Blazers, Liechtenstein). Observations were made with a Quanta 200 MK2 microscope (FEI, Hillsboro, OR, United States) and xT microscope software (FEI). Scanning electron micrographs were used for morphological identification.

### Fluorescent *in situ* Hybridization

Fluorescence *in situ* hybridization was performed to reveal the occurrence and form of prokaryotes on 13 nematodes from station G (2016). Some nematodes were fixed in the field or very soon after sampling. Later, in the laboratory, they were hybridized with universal probes (Eub338 and Non-338) or group-specific probes (Delta495a and EPSY549) (Supplementary Table S2). Whole nematodes were rinsed in a 30% formamide buffer (Durand et al., 2010). They were then incubated in a final volume of 30 μl hybridization buffer containing 30% formamide and 2 μl of each probe at 8 μM for 3.5 h at 46°C. After that, the nematodes were rinsed in a washing buffer for 45 min at 48°C. This step was ended by a final wash in milliQ water at room temperature for 10 min. After a quick drying period, the entire labeled organisms were mounted on a slide in an anti-fade mounting medium (SlowFade^®^ Gold anti-fade reagent, Invitrogen) containing DAPI (4’, 6-diamidino-2-phenylindole), a DNA intercalary agent. Observations were made using an Axio Imager.Z2 microscope (Zeiss, Oberkochen, Germany) equipped with an Apotome.2 slider module (Zeiss) and Colibri.7 light technology (Zeiss) and using an ORCA-Flash4.OLT (Hamamatsu, Hamamatsu-city, Japan) camera. Micrographs were analyzed using Zen (Zeiss) software. In order to observe entire specimen cuticle and organs, bright field and white light were used at different focus plans.

### Phylogenetic Reconstructions

#### *Campylobacterota, Gammaproteobacteria*, and *Zetaproteobacteria* Phylogenies

Eighteen *Campylobacterota-*related sequences (previously known as *Epsilonproteobacteria*), 49 *Epsilonproteobacteria* from GenBank, and 2 outgroups were used in the analysis. The dataset on the 16S rRNA gene was aligned with MUSCLE alignment implemented in Geneious 8.1.9 and then processed in Gblocks© (version 0.91b) to remove gaps (finally leaving 402 bp). Phylogenetic reconstructions were performed with two methods: Bayesian inference (BI) using Mr. Bayes 3.2.6 (Ronquist et al., 2012), and Maximum likelihood (ML) using RAxML BlackBox (Stamatakis et al., 2008) on the CIPRES Science Gateway (Miller et al., 2010). The best-fitting model of evolution was computed with jmodeltest v.2.1.6 (Darriba et al., 2012). Bayesian analysis was carried out with 4 chains of 5 × 10^6^ generations, trees sampled every 500 generations, and a burn-in value set at 25% of the sampled trees. We checked that standard deviation of the split frequencies fell below 0.01 and confirmed convergence of the runs to ensure convergence in a tree search performed using Tracer v1.6. The tree was visualized with FigTree v1.4.3.

Six *Gammaproteobacteria*-related sequences, 50 *Gammaproteobacteria* from GenBank, and 3 outgroups (*Campylobacterota*) were used in the analysis. The final length of the dataset was 426 bp. BI and ML were obtained using the same procedures described above.

Six *Zetaproteobacteria*-related sequences, and 33 *Proteobacteria* from GenBank were used in the analysis. The final length of the dataset was 429 bp. BI was carried out with 4 chains of 10 × 10^6^ generations, with trees sampled every 1000 generations, and ML (with RAxML) were obtained using the same procedures described above.

### *Oncholaimus* sp. Phylogeny

To determine the phylogenetic position of the nematodes, we used two representative sequences, 36 published *Oncholaiminae* and two outgroups (*Bathyeurystomina*). A concatenated alignment of partial 18S and 28S was performed for a final length of 1170 bp (596 bp for 18S, and 574 bp 28S). BI was performed with four chains of 5 × 10^6^ generations, and trees sampled every 100 generations. BI and ML (with RAxML) were obtained using the same procedures described above.

### Data Availability

The data supporting the results of this article are available from the NCBI SRA repository (BioProject PRJNA572561, Accessions SRR10194555–1019624).

All sequences used for phylogenetic analyses are available from GenBank under accession numbers MN496493–MN496496 (18S and 28S) and MN567157–MN567186 (16S bacteria).

## Results

### Morphology and Molecular Identity of the Nematode

**FIGURE 2:**
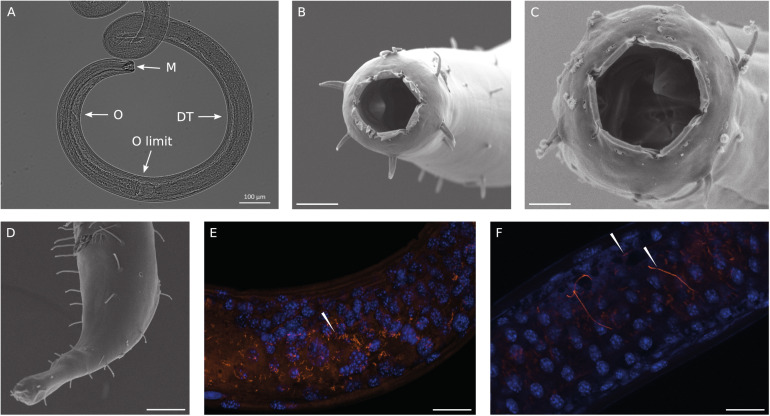
Observations of *Oncholaimus* sp. and associated bacteria. **(A)** Anterior region observed under a stereomicroscope, **(B)** anterior region observed under a scanning electron microscope, **(C)** SEM observation of the buccal cavity, **(D)** SEM observation of the posterior region, **(E,F)** fluorescence *in situ* hybridization observations of bacteria throughout the intestine of the host. In blue, DAPI-stained host nuclei; in orange, bacteria hybridized with the Eub 338 Cy3-labeled probe. Magnification x63. White arrowheads indicate bacteria. Scale bars = 20 μm.

We identified the most abundant nematode recovered in the Gulf of Naples as a new species of *Oncholaimus*. A description is presently being prepared for this species (Zeppilli personal communication), so this will be referred to in the present study as *Oncholaimus* sp. This genus belongs to the more diverse subfamily Oncholaiminae that comprises 11 genera (such as *Oncholaimus* and *Viscosia*). The genus *Oncholaimus* (Smol et al., 2014) is characterized by: left ventrosublateral tooth largest (Figures 2A–C), females monodelphic-prodelphic with antidromously reflexed ovary, demanian system well developed, terminal ducts and pores present in variable number or absent in virgin females, males diorchic, spicules short, gubernaculum absent, tail short (Figure 2D). The species reported in this study is characterized by a cloacal postaperture surrounded by setae and a conical papilla on the tail (Figure 2D).

Phylogenetic reconstruction showed that the investigated nematode specimens from the Gulf of Naples form a well-supported group and belong to a single species (Supplementary Figure S1). The group clustered with three *Oncholaimus* sp. sampled at Appledore, Torridge Estuary, United Kingdom.

### Observations of Bacteria Using SEM and FISH Analyses

SEM analysis revealed the absence of any bacteria settled on the cuticle or in the buccal cavity (Figures 2B–D). The absence of bacteria outside the cuticle was also confirmed using light microscopy at different focus plans (Figure 2A). As observed on Figure 2A, the complete digestive system was observable as the nematode is transparent to light. Thirteen entire specimens of *Oncholaimus* sp. from the Gulf of Naples were used for FISH analyses and showed the occurrence of bacteria. Observations with a general bacterial probe (Eub 338) revealed the presence of three morphologies of bacteria inside the digestive tract: thin filaments (Figure 2E), rod-shaped bacteria and very long filaments (probably long rod chains) (Figure 2F). Rod-shaped bacteria signal decreased along the digestive system from the esophagus toward the posterior part where rod-shaped bacteria were not observable anymore (Supplementary Figure S2). Long filaments, thin or larger ones, showed a high fluorescent signal all along the digestive system (Figures 2E,F and Supplementary Figure S2), which suppose intact and active bacteria (i.e., they had not undergone digestion). Specific probes were also tested (Supplementary Table S2) but no evident signal was obtained. We also checked for autofluorescence and absence of non-specific signal using non-hybridized specimens and a non-sense probe (Supplementary Table S2), as negative controls, confirming our results.

### Negative Controls of Metabarcoding

Three analyses of negative controls were performed with FROGS: one with nematodes plus a DNeasy Blood & Tissue blank, one with sediment plus a DNeasy PowerMax Soil blank, and the third with water plus a DNeasy PowerWater blank. After bioinformatics processing, all OTUs were compared for each dataset. The sediment blank was composed of seven OTUs in common out of 1097 final OTUs; the water blank was composed of 29 out of 492 OTUs and the nematodes blank of 26 out of 822 OTUs. In the overall analysis (three environments at four stations) described below, 14 OTUs from the blank were discarded from the final OTU table. These blank OTUs had an abundance of > 50 reads and were composed of *Actinobacteria* (*Cutibacterium*, *Corynebacterium*), *Firmicutes* (*Staphylococcus*, *Bacillus*), *Gammaproteobacteria* (*Pseudomonas*), and *Betaproteobacteriales* (*Delftia*).

### Bacterial Diversity Analysis

The metabarcoding (region V3-V4 of the 16S rRNA gene) of bacterial communities associated with three environments (Nematode, Sediment, and Water) at three stations (H, G, and Z) (Figure 1) plus the bacterial communities of nematodes for the station H1 of the Gulf of Naples (see Supplementary Table S1 for details) produced 2,752,078 reads after the bioinformatics processing (Supplementary Table S3). The sequences clustered in 1,216 OTUs assigned with the Silva 132 database (Supplementary Table S4). A list with reads count for each samples and all OTUs are given (Supplementary Table S5). Alpha diversity indices values for all environments and stations are shown in Supplementary Figure S3. We observed that richness (number of observed OTUs) from the three environments were significantly different (*p* = 7.399e^–10^, Supplementary Table S6) with lower values for nematodes. The richness was significantly different for the stations only for the nematode (*p* = 0.01124; Supplementary Table S6). Evenness indices (Shannon and Pielou indices) showed the same pattern with significant differences from environments with a high evenness for water and sediment (Supplementary Table S6). More informatively, low evenness combined with low richness indicate a high dominance of a few OTUs such as for bacterial communities of nematodes from G site.

**FIGURE 3:**
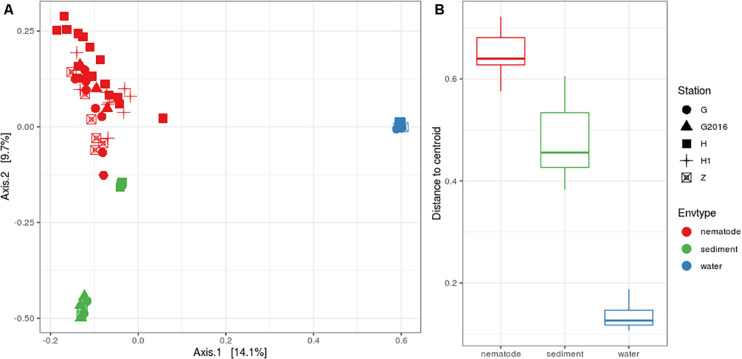
**(A)** PCoA plot based on Bray-Curtis distances illustrating the similarities and differences in the composition of bacterial communities from the three environments at the four stations. **(B)** Distance to centroid from the three environments.

Structure analysis was performed with Beta diversity indices that allowed relationships between bacterial communities to be understood. The PCoA plot showed a clear separation among the three environments but less clear scattering among the stations (Figure 3A). PERMANOVA analyses were significant, suggesting that the “environment” and “station| could explain 22 and 10%, respectively, of the total bacterial variation in the Gulf of Naples. Interactions of “environment” with “station” was also significant and could explain 8%. Pairwise comparisons were significant, for “environment” (i.e., nematode vs. sediment; nematode vs. water; sediment vs. water) but only four pairwise comparisons were significant for “station” (H vs. G-2016; H vs. Z; H1 vs. Z; H1 vs. G-2016) allowing us to group together H and H1 and G and G2016 for further analysis (Supplementary Table S7). Analysis of the beta dispersion (i.e., distance to centroid) showed that microbial community dispersion was higher in nematodes compared to water and sediment, indicating higher beta diversity across these samples (Figure 3B and Supplementary Table S7).

### Microbial Taxonomic Comparison at Three Environments

**FIGURE 4:**
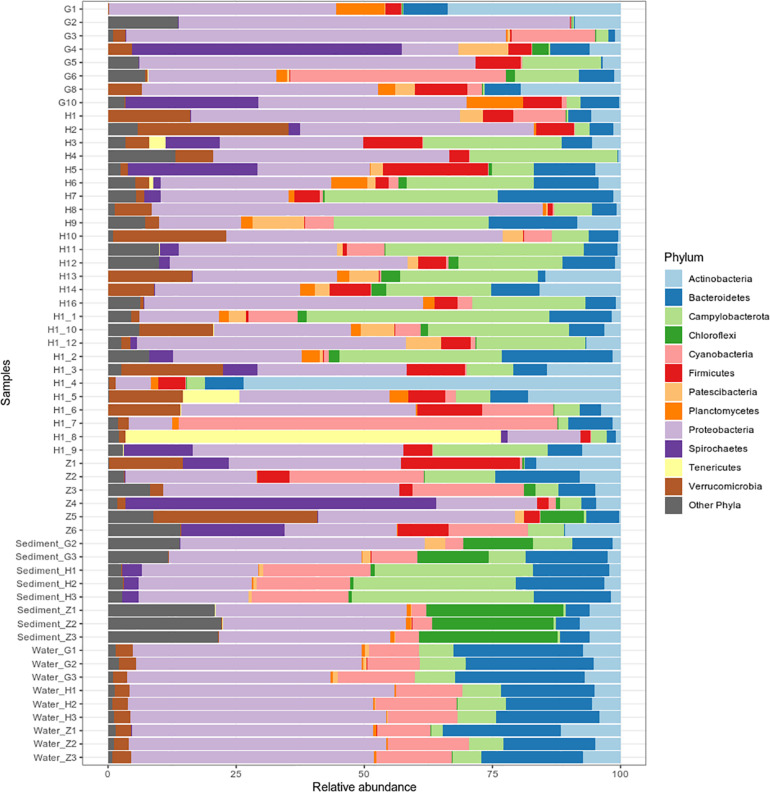
Bacterial community composition of 40 *Oncholaimus* sp. specimens, 8 sediment samples and 9 water samples at the phylum level in 2017. Relative abundance is represented as the percentage of the total effective bacterial sequences per sample. Only phyla representing more than 1% of overall abundance are detailed on the barplot.

**FIGURE 5:**
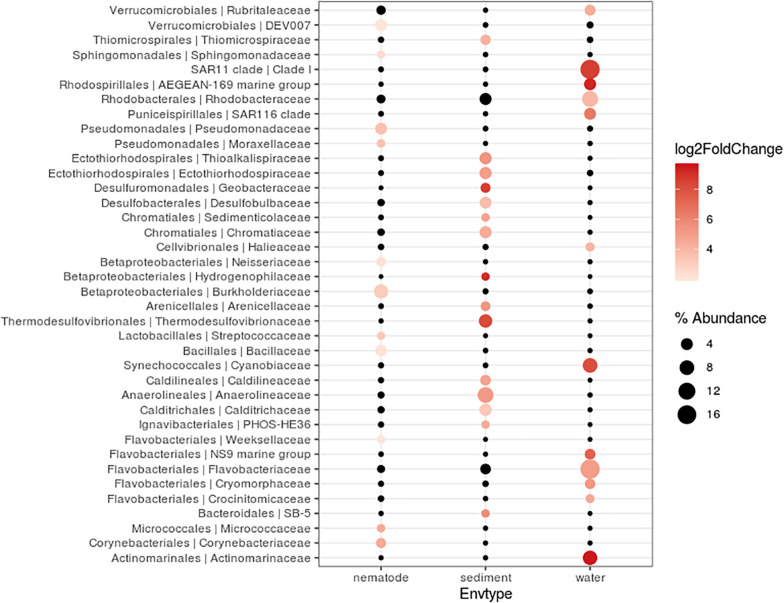
Differentially abundant features at the family level between three environments (nematode, Sediment, Water). Each circle represents a family. Only taxa accounting for more than 1% of the overall relative abundance and overabundant were reported (log2FoldChange > 0). Features significantly overabundant were represented by a gradient of color; non-overabundant features are kept in black. The size of circle is proportional of the percentage abundance.

Bacterial community composition and relative abundance were specific to each environment in 2017: the nematode microbiome was dominated by *Proteobacteria* (37%), *Campylobacterota* (12%), and *Actinobacteria* (9%); the sediment microbial community was dominated by *Proteobacteria* (38%), *Bacteroidetes* (12%), and *Chloroflexi* (11%); and the water microbial community by *Proteobacteria* (47%), *Bacteroidetes* (21%), and *Cyanobacteria* (13%) (Figure 4). A heat map of OTU co-occurrence showed that the repartition of bacteria seem to be more correlated with the factor “environment” than with “station” as all seawater, sediment and nematode samples were each grouped together (Supplementary Figure S4). Indeed, almost all OTUs from water were shared among all stations, but not found in the other environments (sediment or nematode). The same pattern is less obvious for sediment OTUs that seems to be grouped by both factors (environment and stations). A Venn diagram of not rare OTUs (number of reads > 0.1% of total sequences) revealed that no OTUs were shared between water and sediment, only two were shared between water and nematode, and 10 between nematode and sediment (Supplementary Figure 5A). A second Venn diagram revealed that only five bacterial OTUs were shared between the four stations (Supplementary Figure 5B). Stations H and H1 had the greatest number of OTUs in common, whereas Z and G had the least. We conducted a differential abundance analysis of bacterial families to better assess differences among each environment (Figure 5). We observed overabundance of several families characteristic of each environment. For example, the main lineages responsible for the difference of water communities compared to other (i.e., nematode and sediment bacterial communities) seemed to be *Alphaproteobacteria*, especially SAR11 clade, or *Flavobacteriales*. While lineages such as *Thermodesulfovibrionales*, *Deltaproteobacteria* (*Desulfuromonadales*), and *Betaproteobacteriales* (*Hydrogenophilaceae*) were overabundant in sediment bacterial communities. *Gammaproteobacteria* (*Pseudomonales*) and *Betaproteobacteriales* were partly responsible for the nematodes communities difference.

### Bacterial Communities of *Oncholaimus* sp. at Different Stations

**FIGURE 6:**
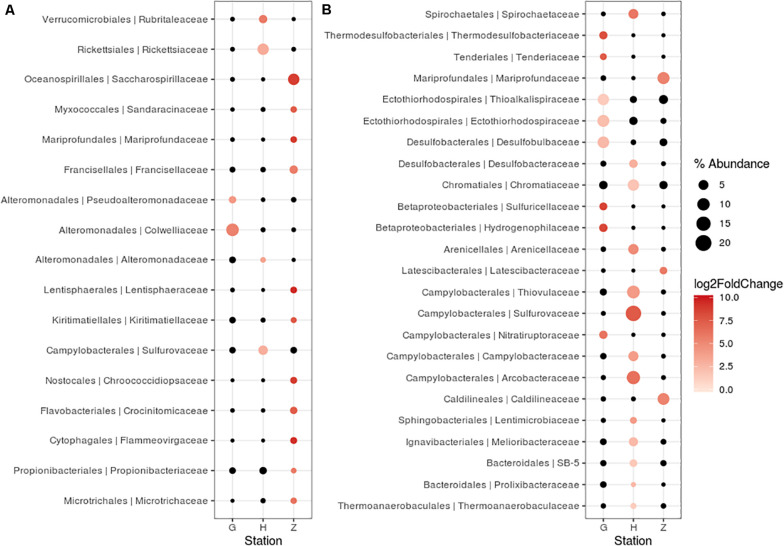
Differentially abundant features at the family level between three stations (G, H, Z). Each circle represents a family. Only taxa accounting for more than 1% of the overall relative abundance and overabundant were reported (log2FoldChange > 0). Features significantly overabundant were represented by a gradient of color; non-overabundant features are kept in black. The size of circle is proportional of the percent of abundance. **(A)** *Oncholaimus* sp. **(B)** sediment.

For nematode bacterial communities analyze, we grouped the G and G2016 results as well as H and H1. A differential taxonomic comparison of nematode at the three stations (G, Z, and H) revealed significant overabundance of some bacterial families (Figure 6A). Station G nematode bacterial communities were mainly related to *Gammaproteobacteria* (*Colwelliaceae* and *Pseudoalteromonadaceae*). For station H, overrepresented OTUs were associated to the occurrence of *Verrucomicrobiales*, *Alphaproteobacteria* (*Rickettsiaceae*), and *Campylobacterota* (*Sulfurovaceae*). Finally, for station Z, they were related to some overrepresented lineages such as *Gammaproteobacteria* (*Oceanospirillales*, *Francisellaceae, Saccharospirillaceae*), *Deltaproteobacteria*, and *Zetaproteobacteria*. Some H specimens also showed the presence of Tenericutes members (Figure 4).

**FIGURE 7:**
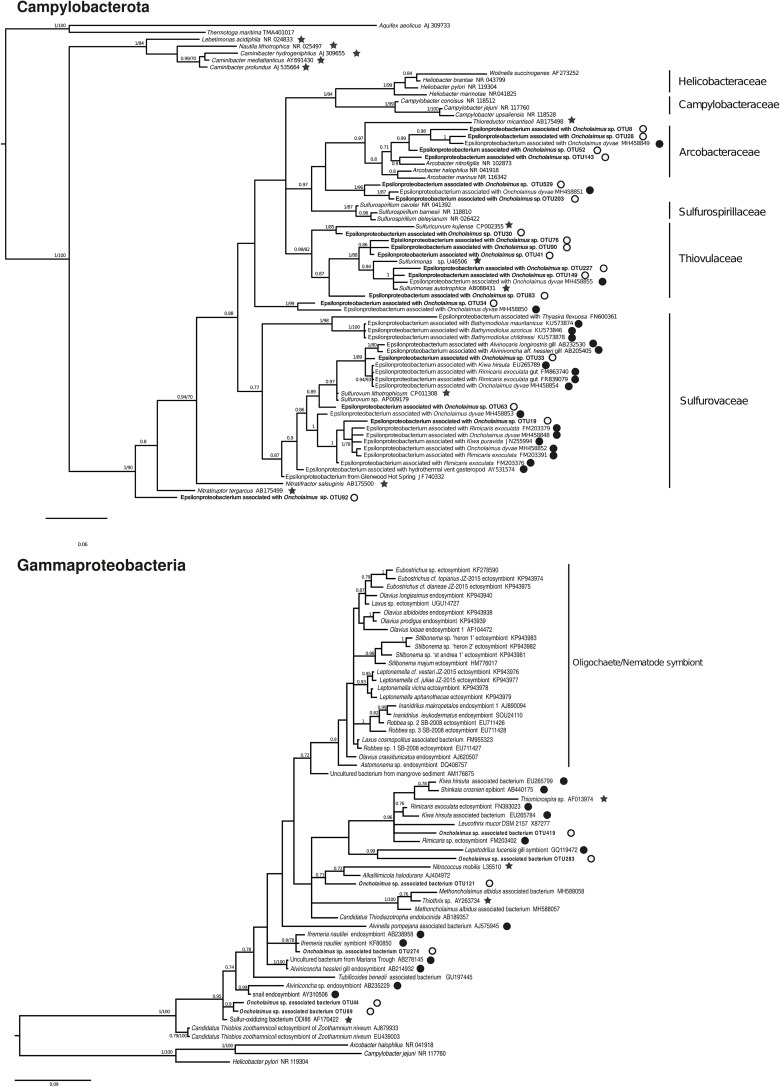
Bayesian inference trees based on the partial 16S rRNA gene for *Campylobacterota* and *Gammaproteobacteria*. The numbers are posterior probabilities (BI) and bootstrap proportions (ML) reflecting clade support (values below 75 are indicated by dashes). Representative sequence names from this study are shown in bold. Black stars indicate an autotroph; white circles a shallow water hydrothermal vent host; and black circles a deep-sea hydrothermal host.

We conducted a more in-depth analysis on the *Campylobacterota* at the three stations. The analysis revealed 59 OTUs split among six genera (*Arcobacter*, *Campylobacter*, *Nitratiruptor*, *Sulfurimonas*, *Sulfurospirillum*, and *Sulfurovum*). For the nematode, only three genera were mainly present at the H station (*Sulfurimonas, Sulfurovum* and *Arcobacter)* and present in G station even if less abundant. Nematode from Z station were deprived of *Arcobacter* relatives, presenting more *Campylobacter* and *Nitratiruptor*. A phylogenetic reconstruction performed with 18 representative sequences of these OTUs plus published sequences affiliated to *Campylobacterota* showed a broad distribution of the new *Oncholaimus* sp. bacterial sequences (Figure 7). The 18 representative sequences were chosen according to their taxonomic affiliation given by BLAST; the unique OTU for the genera *Nitratiruptor*, two OTUs of *Sulfurospirillum*, the most abundant OTU of *Campylobacter*, four more abundant OTUs for *Arcobacter*, three more abundant OTUs for *Sulfurovum*, and six more abundant OTUs for *Sulfurimonas.* The topology of the phylogenetic reconstruction showed that all new 16S rRNA sequences were inserted into a *Campylobacterota* tree and grouped within specific clade such as *Arcobacter*. All representative sequences clustered with sequences from deep-sea hydrothermal vents. Representative sequences belonged to clades within four families (*Arcobacteraceae*, *Sulfurospirillaceae*, *Sulfurovaceae*, and *Thiovulaceae*), and most of them were associated with marine invertebrates in symbiotic relationships.

The diversity of *Gammaproteobacteria* was higher than for *Campylobacterota*, with 258 OTUs identified. A phylogenetic analysis was performed with six representative sequences related to symbionts or sulfur-oxidizing bacteria and many *Gammaproteobacteria* of deep-sea or shallow-water (Figure 7). All endo- or ectosymbionts from marine nematodes (*Astomonema*, *Eubostrichus*, *Laxus*, *Leptonemella*, *Robbea*, and *Stilbonema*) published in GenBank clustered into a large group with other shallow-water organisms, except the new sequences of *Oncholaimus* sp. One new sequence (OTU 419) clustered within a group of deep-sea hydrothermal vent fauna symbionts such as the shrimp *Rimicaris* or the crab *Kiwa*, whereas one other sequence (OTU 274) grouped with the deep-sea hydrothermal vent gastropod *Ifremeria*. Four new sequences were closely related to the sequence of ODIII6, a free-living sulfur-oxidizing *Gammaproteobacteria* obtained from a shallow-water hydrothermal vent in the Aegean Sea (Kuever et al., 2002).

Six OTUs related to *Zetaproteobacteria* were retrieved, which represented 0.5% of total relative abundance (12,412 reads) mainly from sediment and nematodes at station Z. A phylogenetic analysis was performed with these six *Zetaproteobacteria*-related sequences (Supplementary Figure S6). All 16S rRNA sequences clustered into the *Zetaproteobacteria* clade. Two of the sequences from the Gulf of Naples (OTU 125 and OTU 513) formed a clade with an uncultured bacterium from the Loïhi Seamount hydrothermal vent and one (OTU 907) was grouped with a sequence from the Mariana Trough.

### Bacterial Communities of the Sediment at Three Stations (G, H, and Z)

Replicates of each sediment sample showed almost the same lineage distribution in 2017 or 2016 (Figure 4 and Supplementary Figure S7). The overall bacterial community abundance of the sediments associated with the three stations showed the following characteristics: for G, *Proteobacteria*, *Chloroflexi*, and *Bacteroidetes* dominated; for H, *Campylobacterota*, *Cyanobacteria*, and *Proteobacteria* dominated and for Z, *Proteobacteria*, *Chloroflexi*, and *Nitrospirae* dominated (Figure 4). PERMANOVA analyses comparison between stations (G, H, and Z) on sediment microbial diversity showed a global significant difference (Supplementary Table S7). Stations showed different lineage abundances, especially for *Proteobacteria* and *Campylobacterota* phylum (Supplementary Figure S8). *Campylobacterota* were retrieved with almost 80% at H station but less 5% at Z; *Betaproteobacteria* were mostly retrieved at G station (85%) and *Zetaproteobacteria* were dominant at Z station (95%).

A differential taxonomic comparison of bacterial communities of sediment at the three stations revealed significant overabundance at the bacterial families level (Figure 6B). At station Z, *Caldilineaceae* (a thermophilic bacterial family) and *Zetaproteobacteria* (*Mariprofundaceae*, an autotrophic iron oxidizer) were over represented; at station H mainly four lineages of *Campylobacterota* were identified; at station G, lineages of *Gammaproteobacteria* (*Ectothiorhodospriales*), *Betaproteobacteriales*, *Thermodesulfobacteriales* were retrieved. These specific bacterial communities of the sediment at the three stations could be linked to some general observations of the sediment itself (yellow and white mat), its temperature or chemical compounds (Table 2).

**TABLE 2:** Characteristics (depth, water and sediment temperatures, chemistry) of sampling stations.

Station	Depth (m)	T (°C) water	T (°C) sediment (2; 10 cm d.i.s)	CH_4_ (μM) (0; 2; 4 cm d.i.s)	H_2_S (μM) (0; 2; 4 cm d.i.s)	Observations
G (2016)	10.1	72.9 (Geyser fluid)	29.1; ns	Ns	ns	Yellow deposits
G (2017)	10.1	72.8 (Geyser fluid)	ns; 59	0.23; 0.18; 0.16	< 1.5; <1.5; <1.5	Yellow deposits
H	13.5	19.2	26.5; 36.3	0.18; 0.41; 0.46	< 1.5; 2.72; 26.5	White mats
Z	9.7	19.2	33.6; 64	0.38; 0.35; 0.53	< 1.5; <1.5; <1.5	Redsediment
H1	12.1	19.2	21.1; 26	Ns	ns	

**d.i.s, depth in sediment; ns, not sampled.**

### Bacterial Communities of the Surrounding Seawater at Three Stations (G, H, and Z)

The triplicates of the three stations were very close between them (Figure 4), and the overall diversity retrieved on each station was closely related (Supplementary Table S7). Sequences retrieved were mostly *Proteobacteria* SAR11 clade, *Campylobacteria* and marine groups of Bacteroidetes (NS2b, NS4, NS5, NS9) all being clades encountered in seawater bacterioplankton.

### Bacteria Associated With Sulfur, Carbon, Methane, and Iron Cycles

In this study, bacterial lineages known to be involved in the sulfur cycle were identified at different stations or in different environments. The purple sulfur bacteria (PSB), anaerobic bacteria that can use hydrogen sulfide as the electron donor are affiliated to the *Gammaproteobacteria* and divided into two families, the *Chromatiaceae* and *Ecothiorhodospiraceae* (Imhoff et al., 2015). The *Chromatiaceae* contain also non-phototrophs and non-autotrophs bacteria. Six OTUs related to *Chromatiaceae* were identified, which represented 22,847 reads mainly in sediments and nematodes microbial communities at station H. Fourteen OTUs related to *Ecothiorhodospiraceae* were identified, which represented 17,361 reads mostly associated to G sediment. Sulfur-oxidizing bacteria (SOB) affiliated to *Campylobacterota* were detected in this analysis, e.g., genus *Sulfurimonas* (105,203 reads) mainly from H, Z specimens and H sediment or *Sulfurovum* (65,201 reads) from H specimens and sediments. SOB affiliated to *Gammaproteobacteria* were also detected, such as *Thiotrichaceae* (6,519 reads). The largest group of sulfate-reducing bacteria (SRB) includes *Desulfobacterales* and *Desulfuromonadales* among the *Deltaproteobacteria*. Eight OTUs affiliated to *Desulfuromonadales* were retrieved, which represented 14,251 reads mainly in Z sediment, especially the thermophilic genus *Geothermobacter*. Forty-six OTUs related to *Desulfobacterales* were retrieved, divided into two families. *Desulfobacteraceae* (25,693 reads) were detected mainly in H sediment and specimens, whereas *Desulfobulbaceae* (21,635 reads) characterized sediment G. Another group of SRB was found in this study, *Thermodesulfovibrio* (20,461 reads) from the *Nitrospirae* phylum, which was found exclusively in Z and G sediment. Most of these lineages implied in sulfur cycle are autotrophic bacteria, chemosynthetic (such as *Sulfurovum*, *Sulfurimonas*, *Thiotrichacea*) or photosynthetic such as *Ecothiorhodospiraceae* or *Chromatiaceae.* Details (number of reads by samples) are given in Supplementary Table S8.

Potential methanotrophic bacteria, which can obtain their carbon from methane, were detected, with two OTUs related to *Methylococcales* (758 reads) from sediments Z and G. Some sequences related to the phylum *Verrucomicrobia*, which can also oxidize methane (Pol et al., 2007), were identified (107,137 reads), mainly in the water (three stations) and nematodes (four stations) but were absent from sediment samples.

Iron-oxidizing bacteria (FeOB), which play an important role in the iron cycle, are affiliated to *Zetaproteobacteria* (Emerson et al., 2007). In this study, we found six OTUs affiliated to *Mariprofundus* (12,412 reads), mainly in sediment Z and associated with nematodes from Z.

## Discussion

### Shallow-Water *Oncholaimus* sp.-Associated Bacteria

In this study, we found evidence that a free-living marine nematode inhabiting a shallow-water hydrothermal vent field harbors its unique bacterial community. This nematode is deprived of bacteria on the external part of the cuticle but showed intact bacteria all along its digestive system. Microscopic observations (FISH) made it possible to distinguish three morphotypes of bacteria in the digestive cavity, the filament shape being intact all along the digestive system. Additionally, metabarcoding results showed that the bacterial community of *Oncholaimus* sp. was different from its surrounding habitat (water and sediment). Taken together, these results suggest a possible symbiotic relationship, as for the deep-sea nematode *Oncholaimus dyvae* (Bellec et al., 2018). Microbial studies have usually focused on specific endo- or ectosymbionts of one or two marine nematodes, such as well-known examples from the genus *Astomonema* and subfamily Stilbonematinae (Ott, 2004). However, recent research has led to new interest in the study of nematode ecology and global bacterial relationships. A global study of 33 distinct morphological genera showed that nematode microbiome profiles have no distinct patterns across depths, ocean basins or within sites (Schuelke et al., 2018). No correlations could be found among microbiomes, nematode feeding morphology, host phylogeny and morphological identity. Nonetheless, an extended analysis on six Artic sediment sites (i.e., microbial communities of nematodes and sediments obtained from the same core samples) indicated a clear and strong separation among the nematode microbiome and that of the surrounding benthic habitat. Comparable investigations focused on a benthic nematode of the Oncholaimidae family (*Metoncholaimus albidus*) found that specimens harbored distinct microbial communities from the surrounding water, sediment and between different seasons (Bellec et al., 2019). They also showed evidence (microscopic and metabarcoding) of an ectosymbiosis on the cuticle implying *Campylobacterota* and *Gammaproteobacteria* usually associated with invertebrates from deep-sea hydrothermal vents. Here, bacterial phylogenetic trees were constructed and the same pattern was observed. Indeed, in the *Gammaproteobacteria* phylogenetic reconstruction tree, representative sequences were close to *Metoncholaimus albidus* symbionts or to other deep-sea hydrothermal organisms (such as *Rimicaris*, *Kiwa*, *Ifremeria* species). They did not group with other oligochaetes or nematode symbionts like *Olavius*, *Robbea*, *Astonema*, or *Stilbonema*. Lineages related to *Burkholderia* and *Pseudomonas* were also identified. Both lineages are ubiquist heterotrophs and could be part of the nematode microbiome as being able of metal chelation or potential pollutant degradation that could come from both vent fluid emission and organic matter degradation (Lüddeke et al., 2012). Another interesting finding is that two sequences related to a sequence of a free-living sulfur-oxidizing bacterium (Milos strain ODIII6) (Kuever et al., 2002) were retrieved, reinforcing the importance of the sulfur cycle in the nematode microbiome. Regarding the *Campylobacterota* phylogenetic tree, it also showed that most representative sequences clustered with *Campylobacteria* associated to *Oncholaimus dyvae* (Zeppilli et al., 2019) and other deep-sea vent fauna (Dubilier et al., 2008). Indeed, they were mainly grouped within *Arcobacteraceae*, *Sulfurovaceae* and *Thiovulaceae* families (Waite et al., 2017). Detection of *Campylobacterota* associated with nematodes was mainly seen at station H, characterized by a white bacterial mat, H_2_S presence and temperature around 25°C, was therefore consistent (Table 2). Sulfur-oxidizing chemoautotrophic symbionts have already been found associated with *Stilbonema*, *Laxus*, *Astononema*, and *Metoncholaimus* (Nussbaumer et al., 2004; Ott, 2004; Musat et al., 2007; Bellec et al., 2019) in shallow water but also with *Oncholaimus* from deep-sea hydrothermal vents (Bellec et al., 2018). Bacteria of twelve *Oncholaimus dyvae*, a species of Oncholaimidae nematode from the Mid-Atlantic Ridge hydrothermal vent, were characterized and a potential symbiotic role with some sulfur-oxidizing bacteria was suggested (Bellec et al., 2018). Finally, Tenericute relative sequences were also retrieved in some nematode, closely related to deep-sea hydrothermal digestive symbiont (Durand et al., 2010; Apremont et al., 2018) or isopods (Kostanjšek et al., 2007). Still, their role is debated but they seem to be present in digestive systems, associated with fauna living in reduced environments. Then, phylogenetic analyses of nematode microbiome showed closer relationships between bacteria of nematodes from the Gulf of Naples and symbiotic lineages typical of deep-sea hydrothermal vent fauna or free-living bacteria therefore suggesting a lack of correlation between nematode microbiomes and host phylogeny.

Both of these previous publications (Bellec et al., 2018, 2019) describing microbial-associated communities of Oncholaimidae specimens showed an overall stable bacterial community composition across individuals, but variability in the relative abundance of OTUs. These studies focused on a single host at one location, which could explain the weak intraspecific variability. The intra- and interspecific variability of the microbiomes of three cryptic marine nematode species (*Litoditis marina*) showed the existence of species-specific microbiomes and a high intraspecific variability (Derycke et al., 2016). An experiment on offered food source (different bacterial mixtures) demonstrated that morphologically similar species could have different feeding strategies. The present study was focused on one host of the Oncholaimidae family but at different stations with various environmental factors, which could explain the more substantial variability of microbial composition observed. This would support the idea that these nematode-bacterial associations are environment driven rather than being under cophylogenetic mechanisms. More research focused on bacteria as a food source for marine nematodes is essential to deeply understand the intraspecific microbiome and the feeding strategy of the host.

### Shallow-Water Hydrothermal Vent Microbial Communities

It is difficult to discern overall patterns or to make comparisons of microbial communities at shallow hydrothermal vents because most studies have focused separately on a single environment such as sediment, fluid samples or biofilms. In the Mediterranean, microbial studies on sediment from the Aegean and Tyrrhenian Seas have already been conducted. In the Aegean Sea, Milos Island (Greece), an arsenic-rich shallow-water hydrothermal vent was investigated at two sites with similar pH and temperature but different salinities and color mats (Price et al., 2013). In the surface sediments, mainly *Campylobacterota* (*Arcobacter* sp.) and *Bacteroidetes* (*Flavobacteria*) were found at high and low salinity, respectively. In the southern Tyrrhenian Sea, a site named Black Point near Panarea Island in the Eolian Islands (Italy) was explored for sediment and fluid microbial communities (Lentini et al., 2014). Among all samples, *Proteobacteria* (mainly *Alpha*- and *Gammaproteobacteria*) and *Campylobacterota* dominated, followed by *Actinobacteria* and *Bacteroidetes*. Our study was also conducted in the Tyrrhenian Sea but at the more northern site of Pozzuoli Bay (Gulf of Naples) in the largest degassing structure, named “Secca delle Fumose” (Donnarumma et al., 2019). Our metabarcoding results of the 16S rRNA gene on sediment samples at three stations (Z, H, and G) showed large differences in microbial composition and relative abundance of different microbial groups. Bacterial communities of sediments or nematodes at Z station had a specific microbiome, with bacteria related to *Zetaproteobacteria*, a class of iron oxidizing bacteria (Emerson et al., 2007). To date, seven *Zetaproteobacteria*-related strains have been isolated from deep-sea hydrothermal bacterial mats or the estuarine water column (McAllister et al., 2011; Makita, 2018), and 59 OTU have been identified, named ZOTU (McAllister et al., 2019). All *Zetaproteobacteria* known to date are autotrophic, using CBB cycle, iron and/or hydrogen oxidizing bacteria, and suggested to be key players in microaerophilic iron rich ecosystems (Mori et al., 2017; McAllister et al., 2019). At Santorini (Greece) a microbial analysis of the iron- and arsenic-rich sediment of a shallow marine hydrothermal system also reported many *Zetaproteobacteria* (Handley et al., 2010), confirming their potential role in these ecosystems. Yet, none but one *Zetaproteobacteria* is associated to a host, the deep-sea hydrothermal shrimp *Rimicaris exoculata* (Jan et al., 2014; Scott et al., 2015). They are supposed to be implied in host nutrition and detoxification, which may also be the case for the presently studied nematode. Some thermophilic related bacteria (optimum growth temperature of 50°C or above) such as *Thermodesulfovibrio* or *Geothermobacter* were also detected in Z sediment. *Thermodesulfovibrio* is a genus assigned to the *Nitrospirae* phylum and established in 1994 after the isolation of *Thermodesulfovibrio yellowstonii* from a hydrothermal vent in Yellowstone Lake (Henry et al., 1994). All known *Thermodesulfovibrio* (five species to date) are strict anaerobes, capable of reduction of sulfate and thiosulfate with a temperature range for growth from 40–75°C (Frank et al., 2016). Temperatures at different depths in sediment Z were measured and showed a range of 33–64°C that may match with *Thermodesulfovibrio* growth. The genus *Geothermobacter* retrieved at sediment Z belongs to the *Geobacteraceae* family in which some species may be thermophilic and known to be iron (III)-reducing microorganisms. Bacteria in sediment H were mainly composed of *Campylobacterota*, especially *Sulfurovum*. The genus *Sulfurovum* belongs to the *Sulfurovaceae* with a temperature range is 10–40°C (optimum 28–30°C), corresponding to the temperature measured at station H (26.5–36.3°C). Other species and many sequences closely related to this genus have been described from deep-sea, shallow-water hydrothermal vents and with vent fauna suggesting that *Sulfurovum* is common in sulfidic marine environments. Sediment G had a more even microbial composition between *Deltaproteobacteria* and *Gammaproteobacteria*. Many SOB were found, such as *Thiotrichaceae* families, but also many SRB such as *Desulfobulbaceae* and *Nitrospirae*. The genus *Nitratiruptor* was also detected exclusively at G, as was the *Ecothiorhodospiraceae* family, a purple sulfur bacteria (Imhoff et al., 2015). Microbial diversity analysis of sediment G suggest that a high sulfide concentration may be present around the geyser. At the same time, a macrofauna diversity study was conducted on the same sediment samples (G-2016) and pore water chemical analyses were done. Values found in sediment interstitial water showed the presence of sulfide (130.58 ppm) and sulfate (2658.5 ppm) that correlated with our microbial results (Donnarumma et al., 2019). Metabarcoding results from water samples taken at the three stations (Z, H and G) showed a relative low variability across station in microbial composition or relative abundance. No bacterial hydrothermal signature was retrieved in our water samples suggesting that we sampled bottom water rather than fluid water.

## Conclusion

Microscopic observations together with metabarcoding results show that a new shallow-water hydrothermal meiofaunal organism, *Oncholaimus* sp., harbors its own specific bacterial community, distinct from its surrounding environment and characterized partly by the presence of SOB- and SRB-related lineages. Although more studies will have to be conducted, these lineages may be implied in nutrition through chemosynthesis and detoxification for the nematodes. Microbial communities in the shallow-water hydrothermal vent of “Secca delle Fumose” show distinct “signatures” for the three stations (G, H, and Z) for the sediment, but lower variability for the water compartment. SOB and SRB were found at all stations, but with high variability in family composition and relative abundance. Autotrophic *Zetaproteobacteria* known to be involved in iron and sulfur cycles characterized station Z. Station G and H microbial communities seemed to be involved in the carbon cycle, but with different families of SOB, SRB, and PRB, probably according to temperature and chemical gradients, underlying geochemical conditions selection on microbial distribution. The fact that nematode-associated bacteria from shallow vents shows much more similarity with deep-sea vent ones (across invertebrate host taxa) suggest that nematode-bacterial associations may be driven by habitat and environmental drivers rather than co-evolutionary mechanisms. This hypothesis opens new perspectives for future research and brings important questions on the bacterial dispersal between shallow and deep-sea vent systems and the independent development of symbioses in these disparate (bathymetrically and geographically) environments.

## Data Availability Statement

The datasets presented in this study can be found in online repositories. The names of the repository/repositories and accession number(s) can be found at: https://www.ncbi.nlm.nih.gov/genbank/, SRR10194555–1019624.

## Author Contributions

LB, M-AC-B, and DZ analyzed the data and wrote the manuscript. LB carried out the molecular biology experiments and phylogenetic. JA carried out the bioinformatics analysis. LD performed the FISH analysis. NG performed the SEM analysis. CB performed the chemical analysis. RS allowed and helped the sampling at Naples. All the authors read, edited, and approved the final manuscript.

## Conflict of Interest

The authors declare that the research was conducted in the absence of any commercial or financial relationships that could be construed as a potential conflict of interest.

## Footnotes

1 https://dna.macrogen.com

2 http://www.geneious.com

3 www.mrdnalab.com

4 http://bioinfogp.cnb.csic.es/tools/venny/index.html
