# Supplementary material for: Microbial Communities of the Shallow-Water Hydrothermal Vent Near Naples, Italy, and Chemosynthetic Symbionts Associated With a Free-Living Marine Nematode
Source: Front Microbiol. 2020 Aug 20;11:2023. doi: 10.3389/fmicb.2020.02023 (PMC7469538; doi:10.3389/fmicb.2020.02023)
Supplement: Supplementary file 1 [file Table_1.DOCX]

**Supplementary Table S1.** Samples used in this study.

| **Sample ID** | **EnvType** | **Vent site (station)** | **Sampling date** |
| --- | --- | --- | --- |
| H1 | Nematode | H | 11.01.2017 |
| H2 | Nematode | H | 11.01.2017 |
| H3 | Nematode | H | 11.01.2017 |
| H4 | Nematode | H | 11.01.2017 |
| H5 | Nematode | H | 11.01.2017 |
| H6 | Nematode | H | 11.01.2017 |
| H7 | Nematode | H | 11.01.2017 |
| H8 | Nematode | H | 11.01.2017 |
| H9 | Nematode | H | 11.01.2017 |
| H10 | Nematode | H | 11.01.2017 |
| H11 | Nematode | H | 11.01.2017 |
| H12 | Nematode | H | 11.01.2017 |
| H13 | Nematode | H | 11.01.2017 |
| H14 | Nematode | H | 11.01.2017 |
| H16 | Nematode | H | 11.01.2017 |
| H1.1 | Nematode | H1 | 11.01.2017 |
| H1.2 | Nematode | H1 | 11.01.2017 |
| H1.3 | Nematode | H1 | 11.01.2017 |
| H1.4 | Nematode | H1 | 11.01.2017 |
| H1.5 | Nematode | H1 | 11.01.2017 |
| H1.6 | Nematode | H1 | 11.01.2017 |
| H1.7 | Nematode | H1 | 11.01.2017 |
| H1.8 | Nematode | H1 | 11.01.2017 |
| H1.9 | Nematode | H1 | 11.01.2017 |
| H1.10 | Nematode | H1 | 11.01.2017 |
| H1.12 | Nematode | H1 | 11.01.2017 |
| G1-2016 | Nematode | G | 10.12.2016 |
| G2-2016 | Nematode | G | 10.12.2016 |
| G3-2016 | Nematode | G | 10.12.2016 |
| G6-2016 | Nematode | G | 10.12.2016 |
| G1 | Nematode | G | 11.01.2017 |
| G2 | Nematode | G | 11.01.2017 |
| G3 | Nematode | G | 11.01.2017 |
| G4 | Nematode | G | 11.01.2017 |
| G5 | Nematode | G | 11.01.2017 |
| G6 | Nematode | G | 11.01.2017 |
| G8 | Nematode | G | 11.01.2017 |
| G10 | Nematode | G | 11.01.2017 |
| Z1 | Nematode | Z | 11.01.2017 |
| Z2 | Nematode | Z | 11.01.2017 |
| Z3 | Nematode | Z | 11.01.2017 |
| Z4 | Nematode | Z | 11.01.2017 |
| Z5 | Nematode | Z | 11.01.2017 |
| Z6 | Nematode | Z | 11.01.2017 |
| Sediment G1-2016 | Sediment | G | 10.12.2016 |
| Sediment G2-2016 | Sediment | G | 10.12.2016 |
| Sediment G3-2016 | Sediment | G | 10.12.2016 |
| Sediment G2 | Sediment | G | 11.01.2017 |
| Sediment G3 | Sediment | G | 11.01.2017 |
| Sediment H1 | Sediment | H | 11.01.2017 |
| Sediment H2 | Sediment | H | 11.01.2017 |
| Sediment H3 | Sediment | H | 11.01.2017 |
| Sediment Z1 | Sediment | Z | 11.01.2017 |
| Sediment Z2 | Sediment | Z | 11.01.2017 |
| Sediment Z3 | Sediment | Z | 11.01.2017 |
| Water G1 | Water | G | 11.01.2017 |
| Water G2 | Water | G | 11.01.2017 |
| Water G3 | Water | G | 11.01.2017 |
| Water H1 | Water | H | 11.01.2017 |
| Water H2 | Water | H | 11.01.2017 |
| Water H3 | Water | H | 11.01.2017 |
| Water Z1 | Water | Z | 11.01.2017 |
| Water Z2 | Water | Z | 11.01.2017 |
| Water Z3 | Water | Z | 11.01.2017 |
