# Supplementary material for: Microbial Communities of the Shallow-Water Hydrothermal Vent Near Naples, Italy, and Chemosynthetic Symbionts Associated With a Free-Living Marine Nematode
Source: Front Microbiol. 2020 Aug 20;11:2023. doi: 10.3389/fmicb.2020.02023 (PMC7469538; doi:10.3389/fmicb.2020.02023)
Supplement: Supplementary file 2 [file Table_2.DOCX]

**Supplementary Table S2.** Primers and Fluorescent probes used in this study.

| **Gene/Phylotype** | **Primer/Probe** | **Primer/Probe sequence (5’-3’)** | **References** |
| --- | --- | --- | --- |
| 18S rRNA | 18S1.2a | CGATCAGATACCGCCCTAG | (Bernard et al., 2010) |
| 18S rRNA | 18Sr2b | TACAAAGGGCAGGGACGTAAT |  |
| 28S rRNA | D2Ab | ACAAGTACCGTGAGGGAAAGTTG | (Nadler et al., 1999) |
| 28S rRNA | D3B | TCGGAAGGAACCAGCTACTA |  |
| *Eubacteria* | Eub338 | GCTGCCTCCCGTAGGAGT | (Amann et al., 1990) |
| *Deltaproteobacteria* | Delta495a | AGTTAGCCGGTGCTTCCT | (Loy et al., 2002) |
| *Campylobacterota* | EPSY549 | CAGTGATTCCGAGTAACG | (Lin et al., 2006) |
| Nonsense | Non338 | ACTCCTACGGGAGGCAGC | (Wallner et al., 1993) |

Amann, R. I., Krumholz, L., and Stahl, D. A. (1990). Fluorescent-oligonucleotide probing of whole cells for determinative, phylogenetic, and environmental studies in microbiology. *J. Bacteriol.* 172, 762. doi:10.1128/jb.172.2.762-770.1990.

Bernard, E. C., Handoo, Z. A., Powers, T. O., Donald, P. A., and Heinz, R. D. (2010). Vittatidera zeaphila (Nematoda: Heteroderidae), a new genus and species of cyst nematode parasitic on corn (Zea mays). *J. Nematol.* 42, 139.

Lin, X., Wakeham, S. G., Putnam, I. F., Astor, Y. M., Scranton, M. I., Chistoserdov, A. Y., et al. (2006). Comparison of vertical distributions of prokaryotic assemblages in the anoxic Cariaco Basin and Black Sea by use of fluorescence in situ hybridization. *Appl Env. Microbiol* 72, 2679–2690.

Loy, A., Lehner, A., Lee, N., Adamczyk, J., Meier, H., Ernst, J., et al. (2002). Oligonucleotide microarray for 16S rRNA gene-based detection of all recognized lineages of sulfate-reducing prokaryotes in the environment. *Appl Env. Microbiol* 68, 5064–5081.

Nadler, S., Felix, M.-A., Frisse, L., Sternberg, P., De Ley, P., and Thomas, W. K. (1999). Molecular and morphological characterisation of two reproductively isolated species with mirror-image anatomy (Nematoda: Cephalobidae). *Nematology* 1, 591–612.

Wallner, G., Amann, R., and Beisker, W. (1993). Optimizing fluorescent in situ hybridization with rRNA‐targeted oligonucleotide probes for flow cytometric identification of microorganisms. *Cytom. J. Int. Soc. Anal. Cytol.* 14, 136–143.
