# Supplementary material for: Microbial Communities of the Shallow-Water Hydrothermal Vent Near Naples, Italy, and Chemosynthetic Symbionts Associated With a Free-Living Marine Nematode
Source: Front Microbiol. 2020 Aug 20;11:2023. doi: 10.3389/fmicb.2020.02023 (PMC7469538; doi:10.3389/fmicb.2020.02023)
Supplement: Supplementary file 3 [file Table_3.DOCX]

**Supplementary Table S3.** Number of reads (Nb) at different stages of processing.

| **Sample ID** | **Nb after quality process** | **Nb after OTU affiliation** | **Final Nb after filters** |
| --- | --- | --- | --- |
| H1 | 78,105 | 62,357 | 30,613 |
| H2 | 104,137 | 81,459 | 58,547 |
| H3 | 99,379 | 77,782 | 57,759 |
| H4 | 68,990 | 54,737 | 37,071 |
| H5 | 122,966 | 98,182 | 76,333 |
| H6 | 93,762 | 70,826 | 53,971 |
| H7 | 64,478 | 50,035 | 34,711 |
| H8 | 116,024 | 93,980 | 82,540 |
| H9 | 80,114 | 63,762 | 35,572 |
| H10 | 46,961 | 34,634 | 15,702 |
| H11 | 53,161 | 41,608 | 24,402 |
| H12 | 63,438 | 51,545 | 42,760 |
| H13 | 26,426 | 20,957 | 11,697 |
| H14 | 91,550 | 72,148 | 44,552 |
| H16 | 83,757 | 66,742 | 40,222 |
| H1.1 | 96,315 | 74,047 | 66,329 |
| H1.2 | 66,298 | 47,542 | 37,693 |
| H1.3 | 50,573 | 40,199 | 26,171 |
| H1.4 | 78,164 | 61,198 | 57,401 |
| H1.5 | 51,779 | 40,995 | 17,949 |
| H1.6 | 38,034 | 30,194 | 16,537 |
| H1.7 | 77,592 | 58,489 | 52,275 |
| H1.8 | 75,593 | 60,907 | 46,568 |
| H1.9 | 38,896 | 31,340 | 19,235 |
| H1.10 | 36,683 | 29,110 | 20,196 |
| H1.12 | 64,329 | 51,618 | 27,700 |
| G1-2016 | 51,529 | 43,992 | 38,926 |
| G2-2016 | 44,898 | 39,056 | 20,251 |
| G3-2016 | 55,727 | 49,198 | 37,642 |
| G6-2016 | 47,877 | 40,702 | 37,295 |
| G1 | 45,592 | 36,390 | 16,594 |
| G2 | 71,748 | 58,566 | 24,611 |
| G3 | 68,098 | 44,023 | 39,703 |
| G4 | 68,062 | 55,657 | 37,279 |
| G5 | 57,933 | 45,474 | 22,586 |
| G6 | 97,097 | 65,849 | 53,403 |
| G8 | 57,816 | 44,927 | 28,690 |
| G10 | 36,410 | 28,669 | 15,546 |
| Z1 | 77,111 | 60,474 | 39,363 |
| Z2 | 112,591 | 89,086 | 57,023 |
| Z3 | 107,622 | 83,939 | 55,875 |
| Z4 | 80,946 | 67,813 | 56,524 |
| Z5 | 52,885 | 42,650 | 21,097 |
| Z6 | 52,345 | 42,188 | 35,973 |
| Sediment G1-2016 | 62,155 | 28,909 | 27,894 |
| Sediment G2-2016 | 57,163 | 27,360 | 26,894 |
| Sediment G3-2016 | 62,609 | 25,519 | 25,275 |
| Sediment G2 | 81,933 | 56,624 | 49,798 |
| Sediment G3 | 63,112 | 36,836 | 36,189 |
| Sediment H1 | 70,184 | 47,635 | 46,773 |
| Sediment H2 | 75,125 | 50,985 | 50,355 |
| Sediment H3 | 69,533 | 47,257 | 46,287 |
| Sediment Z1 | 73,864 | 43,791 | 43,201 |
| Sediment Z2 | 75,796 | 44,584 | 43,807 |
| Sediment Z3 | 73,436 | 44,606 | 43,917 |
| Water G1 | 91,987 | 70,847 | 64,121 |
| Water G2 | 84,416 | 64,851 | 62,341 |
| Water G3 | 100,882 | 77,210 | 74,743 |
| Water H1 | 101,732 | 79,504 | 72,211 |
| Water H2 | 114,271 | 90,009 | 81,873 |
| Water H3 | 98,719 | 78,448 | 63,167 |
| Water Z1 | 120,133 | 94,046 | 74,988 |
| Water Z2 | 111,387 | 87,208 | 71,830 |
| Water Z3 | 112,012 | 87,785 | 73,507 |
| **Total** | **4759,295** | **3585,060** | **2752,078** |
