# Supplementary material for: Microbial Communities of the Shallow-Water Hydrothermal Vent Near Naples, Italy, and Chemosynthetic Symbionts Associated With a Free-Living Marine Nematode
Source: Front Microbiol. 2020 Aug 20;11:2023. doi: 10.3389/fmicb.2020.02023 (PMC7469538; doi:10.3389/fmicb.2020.02023)
Supplement: Supplementary file 4 [file Table_4.DOCX]

**Supplementary Table S4.** Taxonomies of samples assigned by BLASTn+ using the Silva 132 reference database.

| .**Sample ID** | **Nb phyla** | **Nb class** | **Nb orders** | **Nb families** | **Nb genera** | **Nb species** |
| --- | --- | --- | --- | --- | --- | --- |
| H1 | 24 | 41 | 113 | 180 | 243 | 280 |
| H2 | 26 | 41 | 103 | 164 | 227 | 260 |
| H3 | 28 | 46 | 109 | 174 | 245 | 292 |
| H4 | 23 | 36 | 95 | 152 | 206 | 237 |
| H5 | 27 | 41 | 106 | 172 | 240 | 284 |
| H6 | 30 | 47 | 114 | 179 | 247 | 295 |
| H7 | 32 | 47 | 101 | 158 | 220 | 248 |
| H8 | 26 | 41 | 124 | 198 | 283 | 337 |
| H9 | 25 | 39 | 97 | 154 | 213 | 251 |
| H10 | 23 | 33 | 88 | 134 | 185 | 216 |
| H11 | 29 | 43 | 100 | 147 | 201 | 232 |
| H12 | 28 | 43 | 116 | 185 | 255 | 294 |
| H13 | 21 | 32 | 71 | 104 | 142 | 159 |
| H14 | 28 | 45 | 112 | 178 | 252 | 291 |
| H16 | 31 | 43 | 105 | 162 | 238 | 272 |
| H1.1 | 26 | 41 | 107 | 168 | 255 | 299 |
| H1.2 | 32 | 45 | 111 | 159 | 225 | 257 |
| H1.3 | 21 | 33 | 88 | 145 | 192 | 223 |
| H1.4 | 28 | 46 | 116 | 186 | 262 | 309 |
| H1.5 | 28 | 41 | 85 | 129 | 172 | 200 |
| H1.6 | 24 | 34 | 82 | 113 | 154 | 174 |
| H1.7 | 26 | 43 | 110 | 171 | 236 | 270 |
| H1.8 | 27 | 39 | 94 | 141 | 191 | 222 |
| H1.9 | 23 | 34 | 84 | 118 | 163 | 186 |
| H1.10 | 26 | 38 | 80 | 113 | 157 | 175 |
| H1.12 | 22 | 34 | 97 | 151 | 204 | 240 |
| G1-2016 | 8 | 10 | 21 | 35 | 42 | 48 |
| G2-2016 | 21 | 34 | 86 | 136 | 183 | 205 |
| G3-2016 | 7 | 9 | 21 | 35 | 49 | 55 |
| G6-2016 | 7 | 9 | 23 | 38 | 53 | 60 |
| G1 | 21 | 34 | 86 | 136 | 183 | 205 |
| G2 | 26 | 39 | 105 | 155 | 210 | 250 |
| G3 | 25 | 38 | 100 | 157 | 228 | 272 |
| G4 | 26 | 40 | 108 | 157 | 215 | 243 |
| G5 | 26 | 38 | 98 | 157 | 214 | 248 |
| G6 | 30 | 47 | 119 | 179 | 262 | 303 |
| G8 | 24 | 39 | 101 | 163 | 223 | 250 |
| G10 | 27 | 35 | 82 | 124 | 164 | 183 |
| Z1 | 26 | 44 | 116 | 183 | 268 | 318 |
| Z2 | 32 | 50 | 122 | 195 | 271 | 317 |
| Z3 | 30 | 47 | 118 | 187 | 272 | 322 |
| Z4 | 29 | 43 | 100 | 149 | 203 | 237 |
| Z5 | 28 | 43 | 102 | 146 | 194 | 223 |
| Z6 | 25 | 40 | 100 | 148 | 198 | 229 |
| Sediment G1-2016 | 28 | 48 | 131 | 205 | 309 | 373 |
| Sediment G2-2016 | 32 | 54 | 133 | 208 | 323 | 383 |
| Sediment G3-2016 | 29 | 48 | 130 | 202 | 302 | 364 |
| Sediment G2 | 34 | 57 | 144 | 226 | 348 | 422 |
| Sediment G3 | 34 | 59 | 146 | 211 | 314 | 381 |
| Sediment H1 | 32 | 54 | 135 | 207 | 312 | 373 |
| Sediment H2 | 30 | 52 | 128 | 210 | 326 | 389 |
| Sediment H3 | 33 | 56 | 138 | 216 | 322 | 377 |
| Sediment Z1 | 34 | 57 | 139 | 207 | 314 | 383 |
| Sediment Z2 | 32 | 55 | 143 | 226 | 328 | 394 |
| Sediment Z3 | 33 | 57 | 142 | 214 | 332 | 403 |
| Water G1 | 28 | 43 | 125 | 204 | 311 | 372 |
| Water G2 | 29 | 43 | 113 | 189 | 281 | 339 |
| Water G3 | 30 | 46 | 122 | 202 | 305 | 368 |
| Water H1 | 32 | 49 | 127 | 203 | 298 | 362 |
| Water H2 | 30 | 48 | 129 | 200 | 305 | 372 |
| Water H3 | 29 | 46 | 118 | 193 | 293 | 358 |
| Water Z1 | 30 | 47 | 128 | 214 | 328 | 390 |
| Water Z2 | 27 | 44 | 116 | 190 | 283 | 349 |
| Water Z3 | 31 | 48 | 131 | 209 | 320 | 394 |

Nb = number
