# Supplementary material for: Microbial Communities of the Shallow-Water Hydrothermal Vent Near Naples, Italy, and Chemosynthetic Symbionts Associated With a Free-Living Marine Nematode
Source: Front Microbiol. 2020 Aug 20;11:2023. doi: 10.3389/fmicb.2020.02023 (PMC7469538; doi:10.3389/fmicb.2020.02023)
Supplement: Supplementary file 9 [file Data_Sheet_1.zip › Figure S1.PDF]

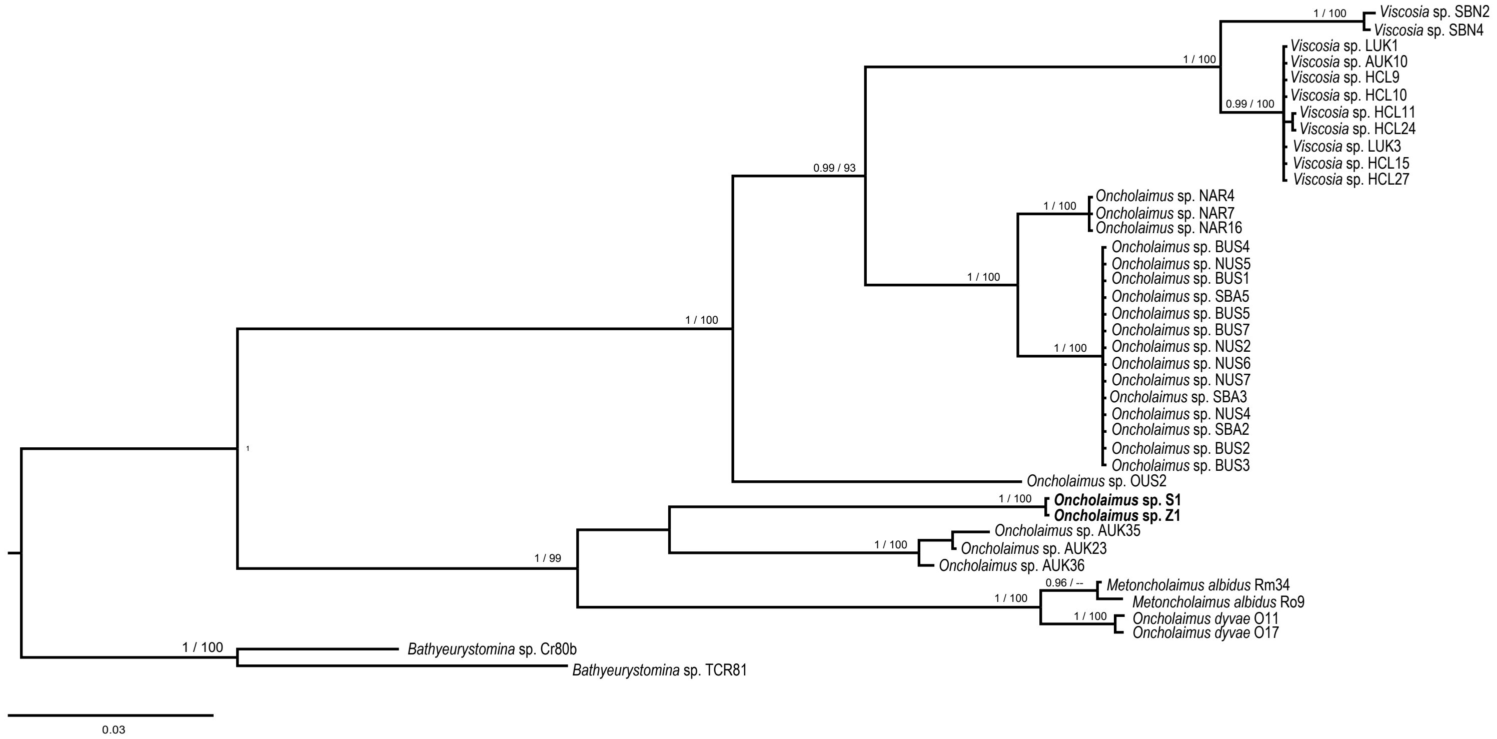

Supplementary Figure S1. Phylogenetic tree of Oncholaiminae based on two partial genes (18S rRNA and 28S rRNA) by BI and ML. The numbers are posterior probabilities (BI) and bootstrap proportions (ML) reflecting clade support (values below 75 are indicated by dashes). Specimens from this study are shown in bold (only two representatives sequences were used). Two *Bathyeurystomonia* were used as the outgroup.
