# Supplementary material for: Microbial Communities of the Shallow-Water Hydrothermal Vent Near Naples, Italy, and Chemosynthetic Symbionts Associated With a Free-Living Marine Nematode
Source: Front Microbiol. 2020 Aug 20;11:2023. doi: 10.3389/fmicb.2020.02023 (PMC7469538; doi:10.3389/fmicb.2020.02023)
Supplement: Supplementary file 9 [file Data_Sheet_1.zip › Figure S2.PDF]

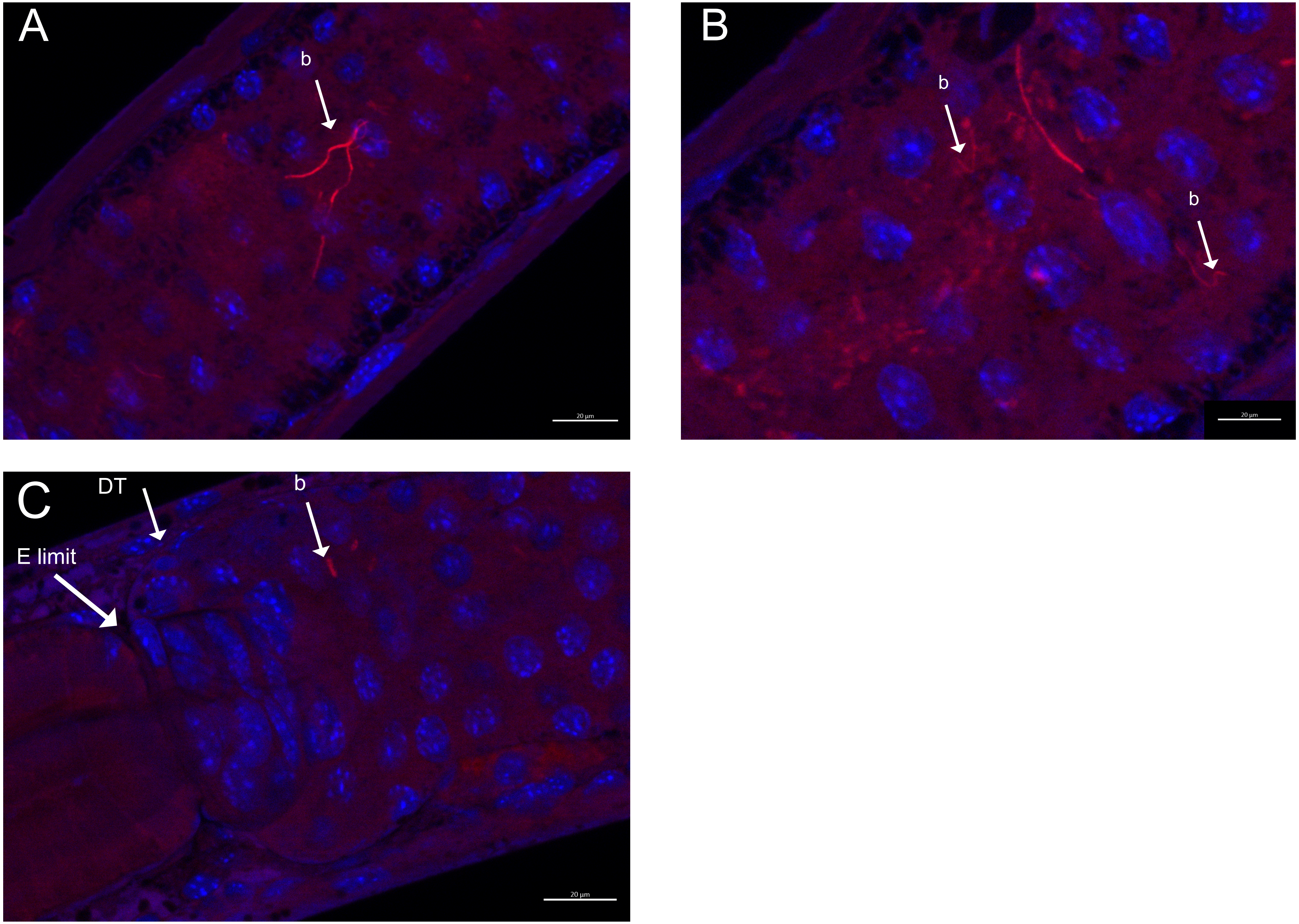

Supplementary Figure S2.

Fluorescence *in situ* hybridization observations of bacteria throughout the intestine of *Oncholaimus* sp.

In blue, DAPI-stained host nuclei; in red, bacteria hybridized by Eub338 Cy5 probe. A) Posterior region, long filaments.;

B) Porterior region, thin filaments. C) Anterior region, rod-shaped bacteria.

Esophagus limit (E limit), Digestive Tract (DT) and Bacteria (b). Magnification x63.
