# Supplementary material for: Microbial Communities of the Shallow-Water Hydrothermal Vent Near Naples, Italy, and Chemosynthetic Symbionts Associated With a Free-Living Marine Nematode
Source: Front Microbiol. 2020 Aug 20;11:2023. doi: 10.3389/fmicb.2020.02023 (PMC7469538; doi:10.3389/fmicb.2020.02023)
Supplement: Supplementary file 9 [file Data_Sheet_1.zip › Figure S5.PDF]

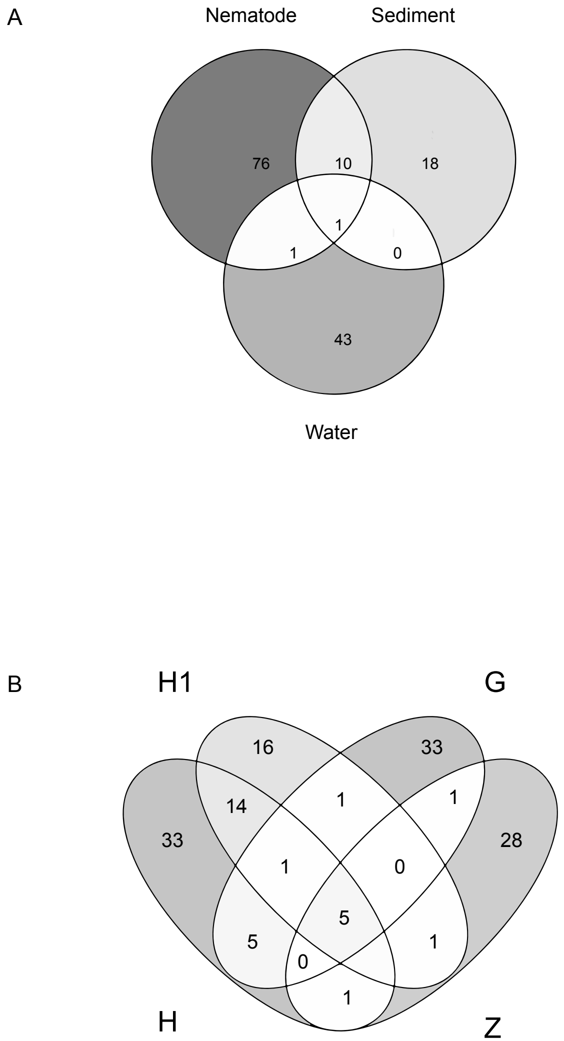

Supplementary Figure S5. Venn diagrams. Only OTUs (> 0.1% relative abundance of total reads) were used. A) between each environment (nematode, sediment and water), B) between the four stations (H, H1, G and Z).
