# Supplementary material for: Microbial Communities of the Shallow-Water Hydrothermal Vent Near Naples, Italy, and Chemosynthetic Symbionts Associated With a Free-Living Marine Nematode
Source: Front Microbiol. 2020 Aug 20;11:2023. doi: 10.3389/fmicb.2020.02023 (PMC7469538; doi:10.3389/fmicb.2020.02023)
Supplement: Supplementary file 9 [file Data_Sheet_1.zip › Figure S6.PDF]

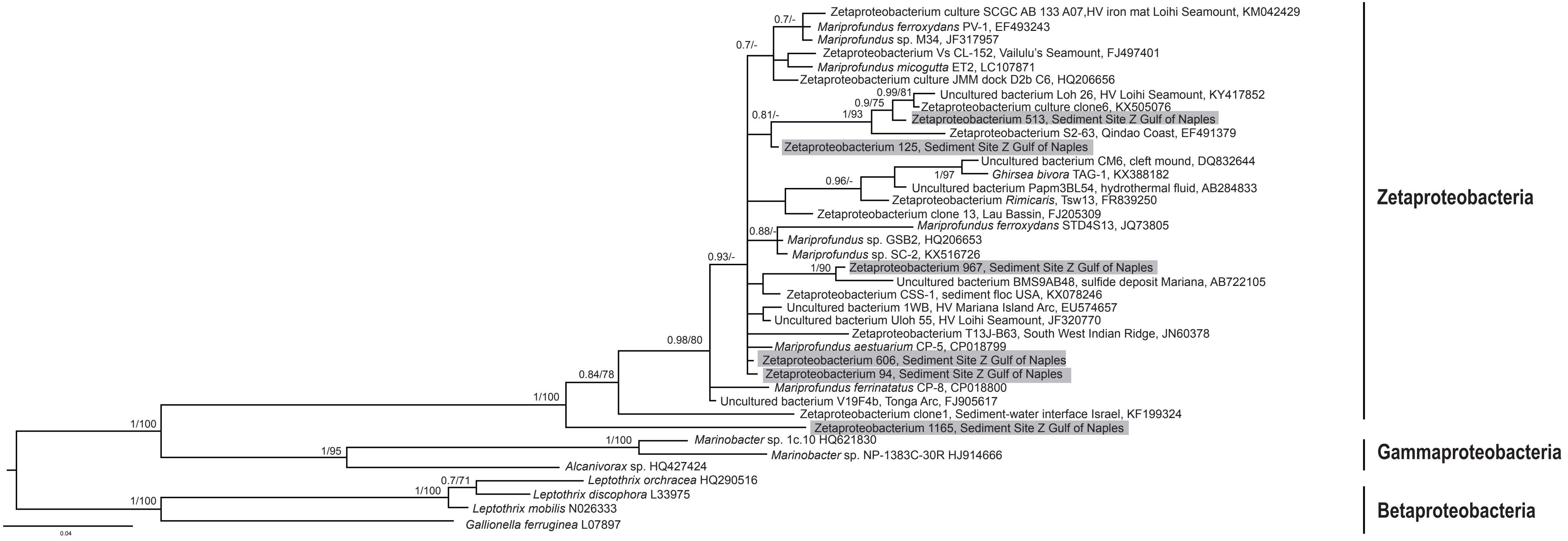

Supplementary Figure S6. Bayesian inference tree based on the partial 16S rRNA gene for *Zetaproteobacteria*. The numbers are posterior probabilities (BI) and bootstrap proportions (ML) reflecting clade support (values below 75 are indicated by dashes). Representative sequences names from this study are shown in grey squares.
