# Supplementary figures and images for: Microbial Communities of the Shallow-Water Hydrothermal Vent Near Naples, Italy, and Chemosynthetic Symbionts Associated With a Free-Living Marine Nematode
Source: Front Microbiol. 2020 Aug 20;11:2023. doi: 10.3389/fmicb.2020.02023 (PMC7469538; doi:10.3389/fmicb.2020.02023)

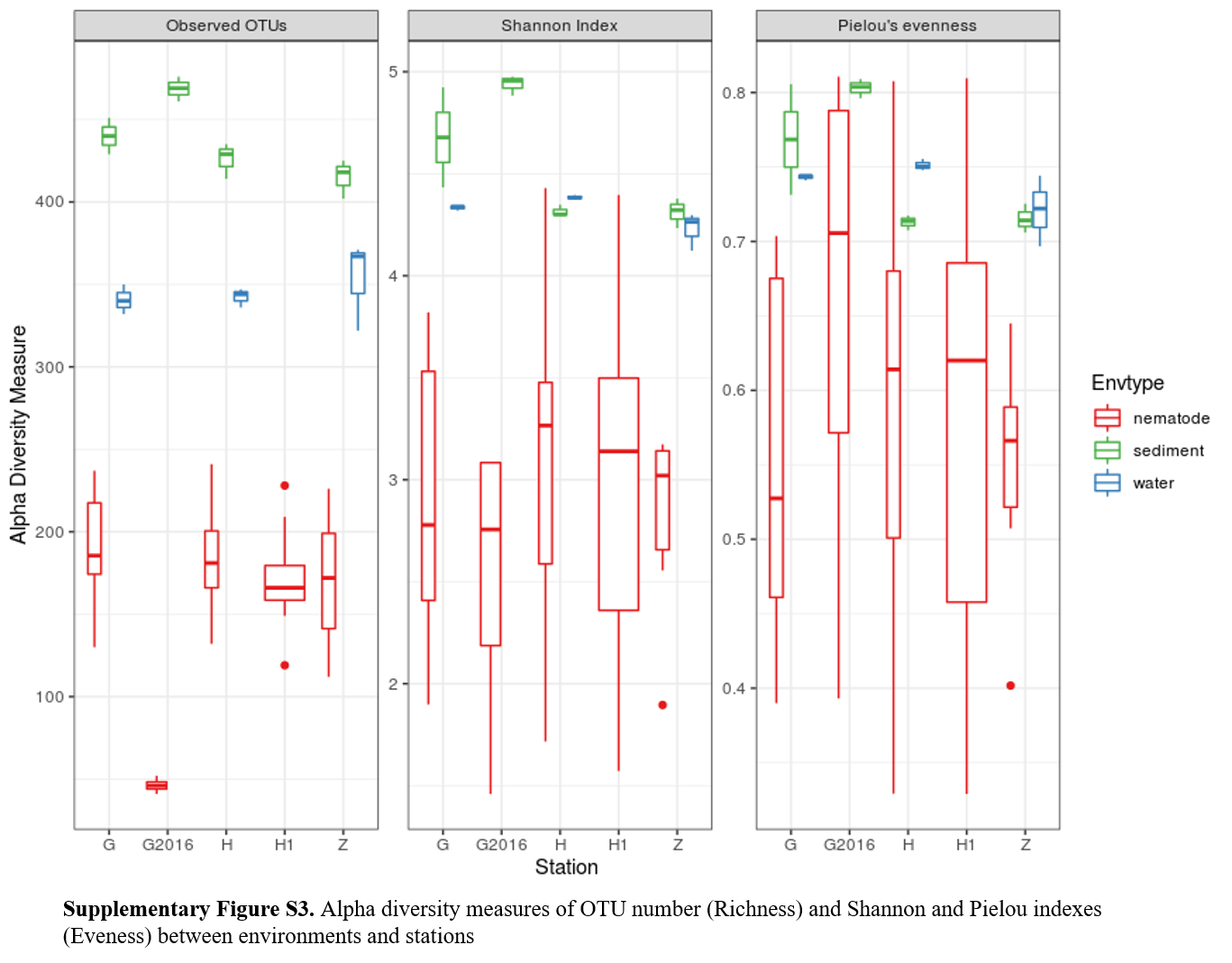

Supplement: Supplementary file 9 [file Data_Sheet_1.zip › Figure S3.TIF]

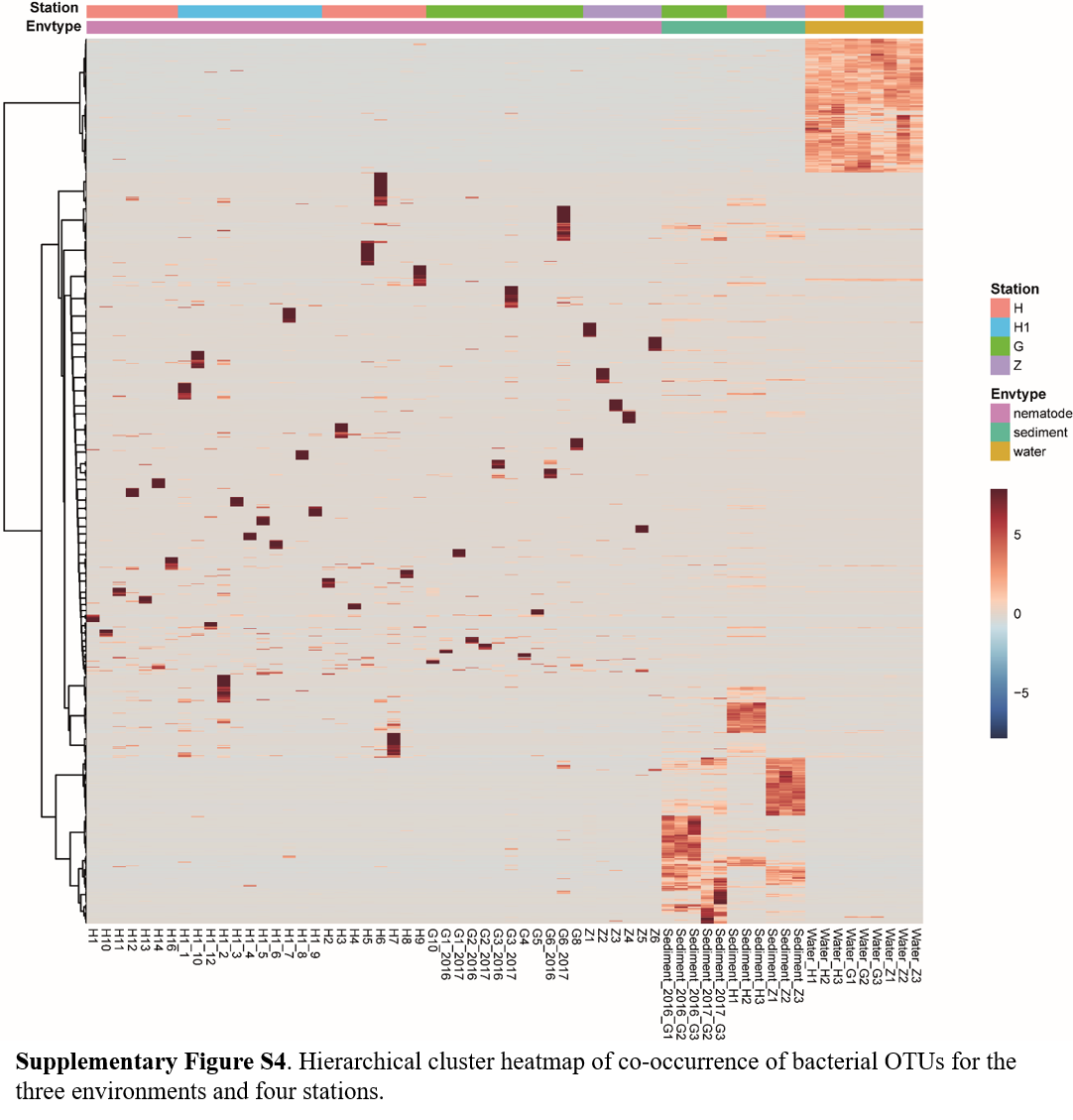

Supplement: Supplementary file 9 [file Data_Sheet_1.zip › Figure S4.TIF]

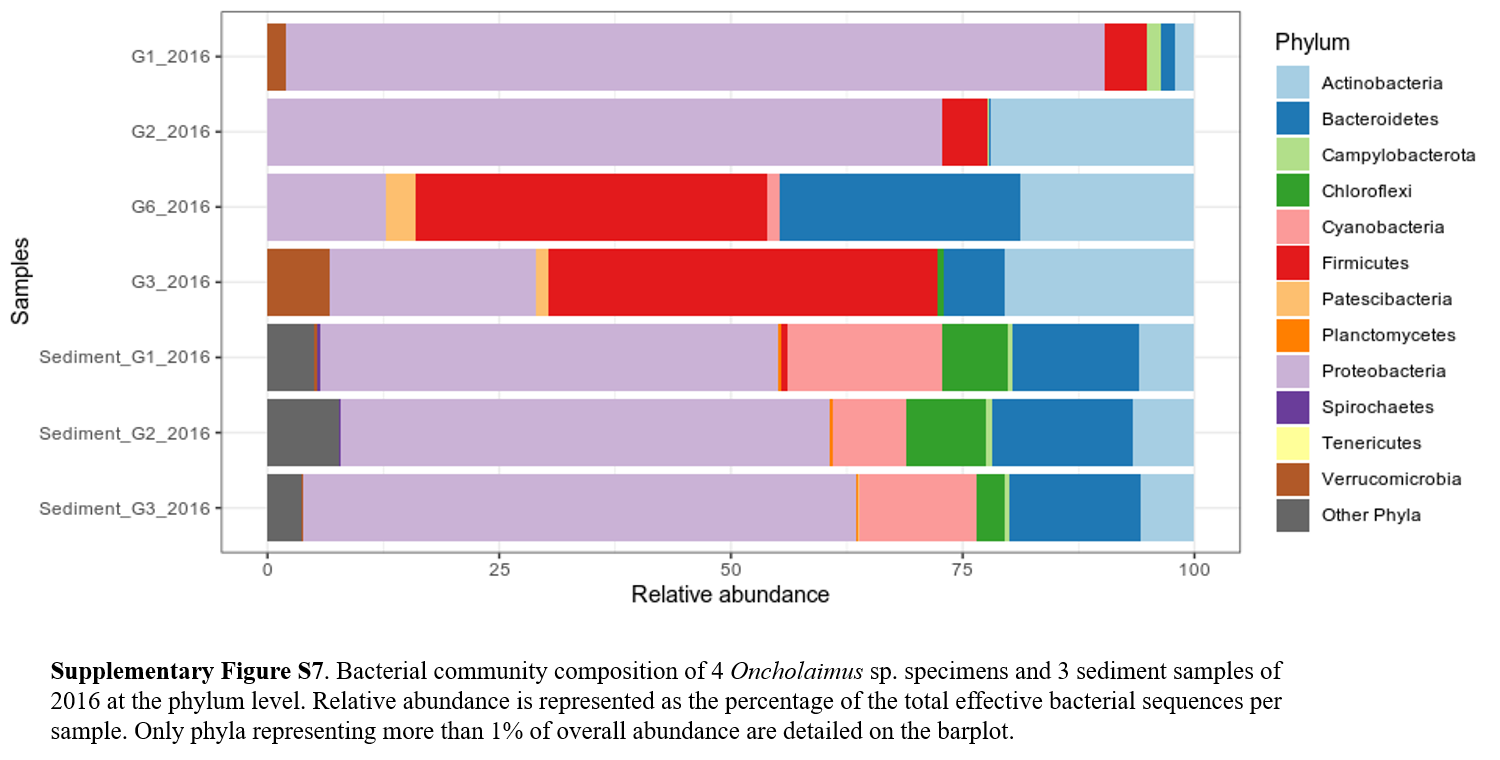

Supplement: Supplementary file 9 [file Data_Sheet_1.zip › Figure S7.TIF]

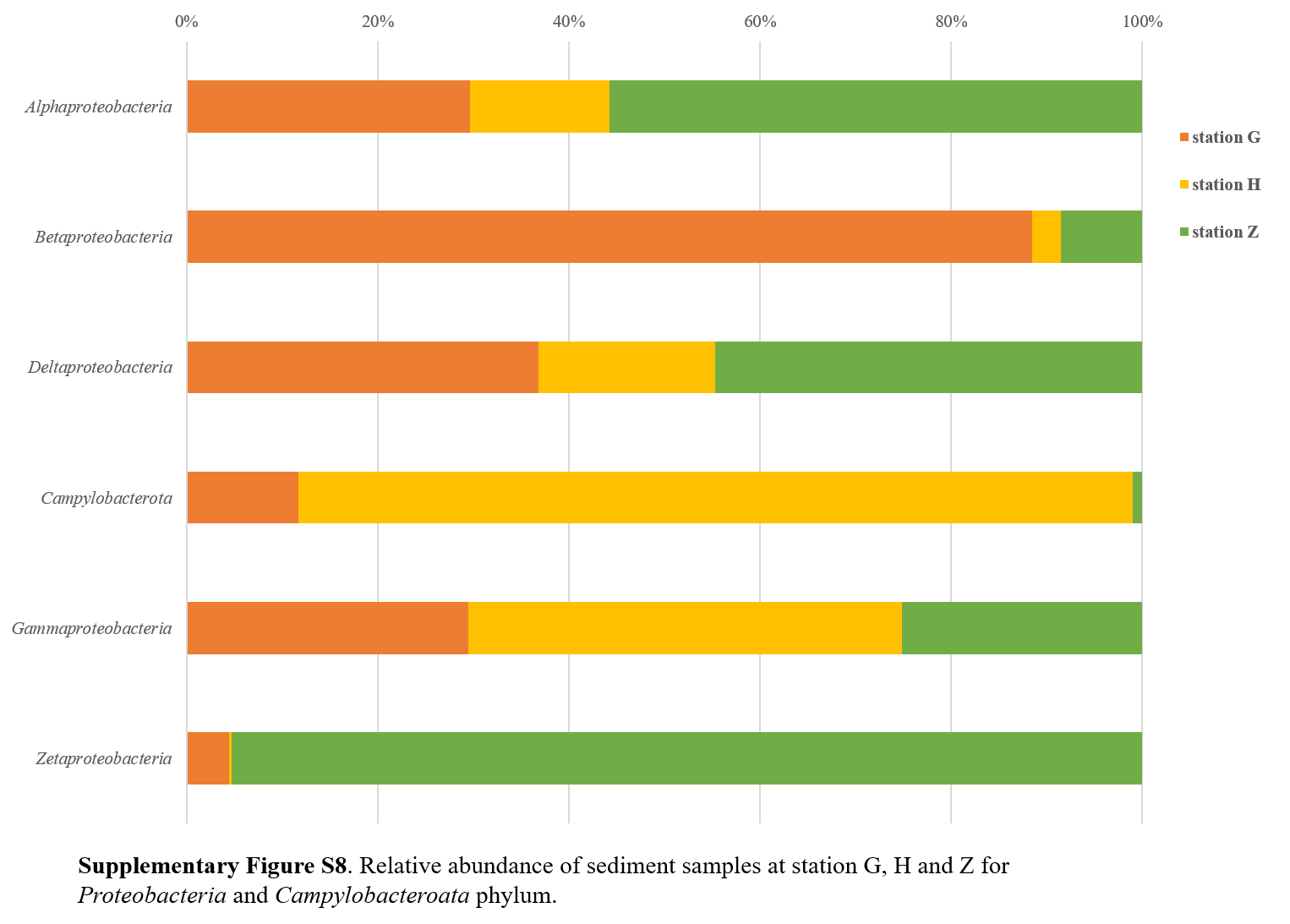

Supplement: Supplementary file 9 [file Data_Sheet_1.zip › Figure S8.TIF]
